# Distinct fitness costs associated with the knockdown of RNAi pathway genes in western corn rootworm adults

**DOI:** 10.1371/journal.pone.0190208

**Published:** 2017-12-21

**Authors:** Ke Wu, Carolina Camargo, Elane Fishilevich, Kenneth E. Narva, Xiuping Chen, Caitlin E. Taylor, Blair D. Siegfried

**Affiliations:** 1 Department of Entomology and Nematology, University of Florida, Gainesville, Florida, United States of America; 2 Dow AgroSciences LLC, Indianapolis, Indiana, United States of America; 3 State Key Laboratory for Biology of Plant Diseases and Insect Pests, Institute of Plant Protection, Chinese Academy of Agricultural Sciences, Beijing, China; University of Tennessee, UNITED STATES

## Abstract

RNA interference (RNAi) based approaches can potentially be used to control insect pests. These approaches may depend on the usage of microRNA (miRNA) or double stranded RNA (dsRNA) mediated gene knockdown, which likely involves proteins that regulate these pathways, such as Argonaute 1 (Ago1), Argonaute 2 (Ago2), Dicer 1 (Dcr1), Dicer 2 (Dcr2), and Drosha in insects. We previously performed functional characterization of *Ago2* and *Dcr2* of western corn rootworm (WCR), *Diabrotica virgifera virgifera* (Coleoptera: Chrysomelidae) and observed that knockdown of *Ago2* and *Dcr2* ameliorated the lethal effect induced by the dsRNA-mediated knockdown of an essential gene in WCR, thereby confirming the involvement of Ago2 and Dcr2 in the dsRNA pathway. In the current study, we identified and characterized additional members of the *Argonaute* and *Dicer* gene families, namely *Ago1*, *Ago3*, *Aubergine*, and *Dcr1*, in a previously developed WCR transcriptome. We also identified a *Drosha* homolog in the same transcriptome. We evaluated the impacts on WCR adult fitness associated with the dsRNA-mediated knockdown of *Ago1*, *Ago2*, *Dcr1*, *Dcr2*, and *Drosha* genes. Among these putative RNAi pathway genes, only the knockdown of *Ago1* incurred significant fitness costs such as reduced survival and oviposition rate, as well as decreased egg viability. The present study, to our knowledge, represents the first report showing that *Ago1* is critical to the survival of insect adults. Our findings suggest that *Ago1* plays an essential role in broader life stages of an insect than previously thought. Importantly, since fitness costs were not observed, downregulation or loss of function of RNAi pathway genes such as *Ago2* or *Dcr2* may confer resistance to pest control measures that rely on the normal functions of these genes. However, the precise roles of these genes under field conditions (i.e., in the presence of possible viral pathogens) requires further investigation.

## Introduction

RNA interference (RNAi) can be broadly defined as regulation of gene expression directed by small non-coding RNA molecules [1]. RNAi can happen at the transcriptional level in the nucleus, via the action of RNA-induced initiation of transcriptional gene silencing complex or at the post-transcriptional level in the cytosol, through the action of the RNA-induced silencing complex (RISC) [2–4].

Nuclear RNAi consists of the Piwi-interacting RNA (piRNA) pathway in which Piwi-like proteins (*e*.*g*., Argonaute 3 and Aubergine/Piwi) bind to diverse piRNAs, silence and immobilize transposable elements in reproductive organs through either transcriptional suppression or the establishment of methylation patterns [5–10]. Piwi-like proteins belong to the Piwi subfamily of the Argonaute (Ago) protein family.

Cytosolic RNAi includes two parallel, yet partially overlapping, pathways: the double-stranded RNA (dsRNA) and microRNA (miRNA) silencing pathways [11–14]. The dsRNA pathway is induced by endogenous or exogenous dsRNA that are recognized by Dicer, a RNase III protein, which cuts these molecules into double stranded small interfering RNAs (siRNAs) of 21–23 nucleotides [15]. Using siRNAs as guides, RISCs identify target mRNAs that are cleaved by RISC-bound Argonaute, achieving post-transcriptional gene silencing [12, 16–18]. The miRNA pathway involves endogenous miRNAs that are processed by the RNase III-like enzyme Drosha in the nucleus and a Dicer protein in the cytosol. The mature miRNAs are loaded onto an Argonaute family protein and together, they regulate the expression of target mRNAs by blocking ribosomes and reducing mRNA translation elongation. The miRNA-Ago complex may also promote mRNA decay by affecting the polyadenylation status of the mRNAs [19, 20].

Argonaute proteins, initially identified in plants, were later found in some prokaryotes and ~65% of sequenced eukaryotic genomes including fission yeast, plants, invertebrate and vertebrate animals [21–23]. In *Drosophila*, two Argonaute proteins, Argonaute 1 (Ago1) and Argonaute 2 (Ago2), are involved in the miRNA and dsRNA pathways, respectively. Argonaute proteins are characterized by the presence of conserved PAZ (Piwi-Argonaute-Zwille) domain that is involved in dsRNA binding and PIWI (P-element induced wimpy testis) domain that possesses RNase activity [24]. A study in insects found that *Ago1* and *Ago2* transcripts are present in 67 and 94 of 100 surveyed transcriptomes, respectively [25].

Dicer proteins are also widely present in eukaryotic organisms such as plants, fungi, and animals [22, 26]. Dicer proteins contain several conserved domains that encode for functions such as RNA binding, helicase and ribonuclease activities [15]. Unlike some animals (*e*.*g*., *Caenorhabditis elegans*) that utilize one Dicer protein for the production of both miRNAs and siRNAs, *Drosophila* uses Dicer 1 (Dcr1) and Dicer 2 (Dcr2) in the processing of miRNAs and siRNAs, respectively [27]. In insects, transcripts of *Dcr1* and *Dcr2* orthologs have been found in 66 and 80 of 100 investigated transcriptomes, respectively [25].

Drosha proteins, while absent in plants, are widely distributed in animals from nematodes to humans [22, 26]. In insects, *Drosha* transcripts have been found in 79 of 100 transcriptomes evaluated [25]. Drosha proteins contain a conserved domain (RIBOc) that is also present in Dicers and a domain responsible for cleaving dsRNA [28].

In *Drosophila*, loss of *Ago2* or *Dcr2* abolishes dsRNA-mediated gene silencing, but does not strongly affect development or physiology [29]. In contrast, *Ago1* mutations result in a multitude of defects, including impaired oocyte development, loss of germline stem cells in adults, defective neural development and lethality in embryos [30–33]. *Drosophila Dcr1* and *Drosha* mutants display impaired oocyte formation and reduced germline cell division as well, suggesting that Ago1 protein and its miRNA biogenesis partners are important for oogenesis and germline cell division in this model insect [31].

Gene silencing pathways involving siRNA and miRNA can potentially be exploited to control agricultural insect pests such as western corn rootworm (WCR), *Diabrotica virgifera virgifera* LeConte, a major insect pest of corn in the United States. Western corn rootworm presents a particular challenge to manage due to its ability to evolve resistance to insecticides, including *Bacillus thuringiensis* toxins expressed by transgenic corn plants [34–37]. Alternative pest management strategies have been proposed to treat insect pests that are prone to develop resistance to conventional control measures, including the use of transgenic plants expressing dsRNA or artificial miRNA that are specific to essential genes of insect pests. The proof of principle for these strategies has been obtained after a number of studies show that transgenic plants expressing insect-specific dsRNA or miRNA can be effective in managing insect pest species, including WCR [38–41]. Reflecting a growing acceptance of RNAi-based pest control technology, the first transgenic maize plants that express dsRNA targeting a WCR essential gene have recently gained approval by multiple regulatory agencies (*i*.*e*., EPA, FDA, and USDA) in the U.S. (https://www.epa.gov/pesticide-registration/epa-registers-innovative-tool-control-corn-rootworm) and have been recognized by an international organization that promotes the world-wide use of crop biotechnology (http://www.isaaa.org/gmapprovaldatabase/event/default.asp?EventID=367).

For an effective management of WCR using RNAi-based strategy, it is important to identify and characterize the components of the dsRNA and miRNA gene silencing pathways. A previous study showed that silencing of *Ago2* and *Dcr2* reduced the effects caused by dsRNA-mediated silencing of critical pigmentation/tanning genes [42], suggesting that Ago2 and Dcr2 proteins are likely components of the dsRNA gene silencing pathway. A more recent study showed that suppression of *Ago2* and *Dcr*2 genes resulted in reduced mortality as well as reduced *vATPase A* knockdown after subsequent exposure to lethal concentrations of *vATPase A* dsRNA [43]. These results raise the possibility that the effectiveness of dsRNA-based pest control measure may be reduced if the expression of *Ago2* or *Dcr2* is impaired in WCR. However, it is unclear what effect downregulation of RNAi pathway genes such as *Ago2* and *Dcr2* may have on WCR fitness. It is also unknown whether WCR possesses other putative RNAi pathway genes such as *Ago1*, *Dcr1*, or *Drosha*, and whether knockdown of these genes would affect fitness.

In the current study, we identified the transcripts of additional *Argonaute* gene family members, including *Ago1*, *Ago3*, and *Aubergine*, in a WCR transcriptome. We also identified *Dcr1* and *Drosha* transcripts in the same transcriptome. In addition, we evaluated the effects of gene knockdown of *Ago1*, *Ago2*, *Dcr1*, *Dcr2*, and *Drosha* on WCR adult fitness. We found that knockdown of *Ago1*, but not *Ago2*, *Dcr1*, *Dcr2*, or *Drosha* had significant fitness costs that were assessed by comparing the viability, oviposition, and egg hatch rates.

## Materials and methods

### Identification, annotation, and phylogenetic analyses of *Argonaute*, *Dicer*, and *Drosha* transcripts

To identify putative WCR *Argonaute* transcripts, tBLASTn searches, using the protein sequences of *Ago1*, *Ago2*, *Ago3*, *Piwi*, and *Aubergine* from *D*. *melanogaster* and *Tribolium castaneum* as queries, were performed on a WCR transcriptome that was assembled from pooled datasets from eggs, neonates, and the midguts of third instars [44, 45]. Iterative searches were conducted with each newly identified WCR *Argonaute* transcript as query until no new transcripts were identified in each subfamily. Putative *Dicer* and *Drosha* transcripts were identified in a similar manner using the protein sequences of *Dcr1*, *Dcr2*, and *Drosha* of *D*. *melanogaster* and *T*. *castaneum* as queries.

The initial annotations of *Argonaute*, *Dicer*, and *Drosha* transcripts were performed based on their homology with well-studied query sequences from *D*. *melanogaster*. These annotations were further verified by performing reciprocal BLASTp searches, using the deduced amino acid sequences of WCR *Argonaute*, *Dicer*, and *Drosha* transcripts, against databases from which query sequences were derived. For further validation, the deduced amino acid sequences of these transcripts were used to search the conserved domain database (CDD: http://www.ncbi.nlm.nih.gov/Structure/cdd/wrpsb.cgi) to ascertain whether they contain the canonical domains for each type of protein (Domain ID for Ago1/Ago2: cd04657, cd02846/cd02825, and pfam08699; Domain ID for Ago3/Aubergine/Piwi: cd04658, smart00949, and cl24903; Domain ID for Dcr1/Dcr2: cd15903, pfam00271/smart00490/smart00949, pfam03368, cd02843/smart00949, cd00593, cd00048/pfam00035/cl00054, and cd00046; Domain ID for Drosha: cd00593 and cl25983).

For the phylogenetic analysis of *Argonautes*, the deduced amino acid sequences of the PIWI domains of WCR *Argonautes* were aligned with those of corresponding homologs from *D*. *melanogaster* and *T*. *castaneum* using MAFFT 7.147 [46] with the E-INS-i alignment algorithm and the BLOSUM 62 matrix. A midpoint-rooted trees was constructed using a maximum likelihood approach with PhyML3.0. [47]. The maximum likelihood tree of WCR Dicers and Drosha was constructed in a similar manner based on the alignment of the full-length amino acid sequences of *Dicers* and *Drosha* from WCR, *D*. *melanogaster*, and *T*. *castaneum*.

The nucleotide and deduced amino acid sequences of WCR *Argonautes*, *Dicers*, and *Drosha* were deposited in GenBank and their available accession numbers are provided in S1 Fig.

### RNA extraction and cDNA synthesis

RNA from different life stages of WCR was extracted using the RNAqueous^®^-Micro kit (Part Number Am1931, Life Technologies, CA, USA) according to the manufacturer’s instructions. cDNA was made using the cloned AMV first-strand cDNA synthesis kit (cat. no. 12328–032, Invitrogen, CA, USA) according to the manufacturer’s instructions. One microgram of total RNA isolated from a pooled sample of WCR (2 females, 2 males, and 5 eggs, 5 larvae) was used in a 20-μl reverse transcription reaction containing the manufacturer’s recommended ingredients including Oligo(dT)_20_ primers. The reaction was performed in a thin-walled tube using a thermocycler (C1000 Touch^™^ Thermal Cycler, BIO-RAD). The reaction was incubated at 50°C for 60 min, followed by incubation at 85°C for 5 min.

cDNA for quantitative reverse transcriptase PCR (qRT-PCR) was made using high-capacity cDNA reverse transcription kits (part number 4375575, Applied Biosystems, CA, USA) according to the manufacturer’s instructions. Five hundred nanograms of total RNA from individual WCR females was used in a 20-μl reaction containing all ingredients. The reaction was incubated at 25°C for 10 min, then 37°C for 120 min, and 85°C for 5 min. Forty μl of TE (10 mM Tris pH 7.5, 1 mM EDTA) was then added to the cDNA reaction (1: 3 dilution). cDNA synthesized by both methods was stored at -20°C until use.

### dsRNA synthesis

Primers with the T7 promoter sequence added at the 5’ ends (S1 Table) were used to amplify fragments (~ 500 bp) of *Ago1*, *Ago2*, *Dcr1*, *Dcr2*, *Drosha*, and *GFP* genes that were later used for dsRNA synthesis. PCR products for WCR genes were amplified from 1 μl of cDNA prepared as described above. PCR products for *GFP* gene were amplified from 50 ng of pGLO plasmid (Cat. No. 1660405, BIO-RAD). Bands of the expected size (~ 500 bp) were extracted and purified using a Gel Extraction kit (Qiagen, Valencia, California) according to the manufacturer’s protocol. Direct sequencing of the purified PCR products was performed at the Interdisciplinary Center for Biotechnology and Research at the University of Florida using the primers used for PCR amplification. dsRNAs were synthesized from 1 μg of purified PCR products (500 bp) using MEGAscript RNAi kit (cat. No. AM1626, Life Technologies, CA, USA) according to the manufacturer’s instructions. Sizes of purified dsRNAs were confirmed by gel electrophoresis on a 1% agarose gel containing 1X TBE buffer. Concentrations of purified dsRNAs were determined by spectrophotometry (NanoDrop 1000, Thermo Scientific, USA), and purified dsRNAs were stored in elution buffer at -20°C until further use.

### Loss-of-function analyses

Newly emerged, non-diapausing WCR adults (mixed sexes), purchased from Crop Characteristics Inc. (Farmington, MN), were provided with fresh sweet corn ears and allowed to mate for at least 4 days before bioassays. Western corn rootworm females (5–6 days old and in groups of 10) were transferred into 118 ml soufflé cups and starved for 24 hrs. They (except water control WCR) were then fed with a total of 10 μg of dsRNA per WCR over a 12-day period (2 days of sucrose + dsRNA and 10 days of artificial diet + dsRNA). For the first 2 days of the 12-day period, the WCR females were provided with 83.3 μl of solution containing 300 ng dsRNA/μl in 20% sucrose (Sigma) each day. Water control females were provided with 83.3 μl of 20% sucrose solution. For the next 10 days of the 12-day period, WCR females were provided with five artificial diet plugs (~ 4 mm in diameter and 2 mm in height) each coated with 20 μl of dsRNA (100 ng/ μl) or water (for water control females) every other day. The artificial diet was prepared as described previously [44].

After the initial 12-day treatments, females (in groups of 5) were transferred to polystyrene oviposition boxes (7.5 cm × 5.5 cm × 5.5 cm) (ShowMan box, Althor Products, Wilton, CT) and were evaluated for their fitness on short-term (4 days) or long-term (28 days) basis with 5 females in each oviposition box representing one biological replicate. For the short-term (4 days) loss-of-function studies of *Ago1* and *Ago2*, females in each oviposition box were provided with 2 diet plugs treated (in the same manner as above) with water or dsRNA every other day. For the long-term (28 days) loss-of-function study of *Ago2*, a different cohort of WCR females underwent the initial 12 days of exposure to various dsRNA or control treatments in the same manner as in the short-term study. They were then allowed to oviposit for 7 days after being moved to oviposition boxes. During this 7 day-period, water- or dsRNA-treated diet plugs were provided every other day. After the initial 7-day period, females were transferred to new oviposition boxes every 7 days and water or dsRNA-treated diet plugs were provided only once a week for 3 weeks. Untreated diet was provided every other day during the last three weeks of the oviposition period.

The fitness parameters measured during the loss-of-function studies included the survival and oviposition rates, and the hatch rate of the eggs. The fitness evaluation was performed using the design of a previous study [48]. The oviposition boxes contained moistened silty clay loam soil that was pre-sifted through a 60-mesh sieve and autoclaved (Jackson, 1986). The oviposition boxes were held at 25 ± 1°C, RH > 80% and L:D 16:8.

At the end of the short-term (or long-term) loss-of-function studies, the surviving WCR were counted and then removed from oviposition boxes, with one female (representing one biological replicate) from each oviposition box processed for expression analyses by qRT-PCR.

The short-term loss-of-function study of *Dcr1*, *Dcr2*, and *Drosha* was performed in the same manner as *Ago1* and *Ago2*. On day 4 of the short-term study, the numbers of surviving WCR were recorded and one WCR from each oviposition box was collected for gene expression assay. The remaining WCR females were evaluated for another 24 days. The WCR females were pooled in groups of five, transferred into new oviposition boxes, and provided with water- or dsRNA-treated diet. They were moved to new oviposition boxes again on days 7 and 14 after the start of the 24-day assay and provided with water- or dsRNA-treated diet plugs on days 3, 10, and 18.

Eggs deposited during the oviposition period were incubated in soil within the oviposition boxes for 10 days at 25°C in the dark before being removed from the soil by washing through a 60-mesh sieve. Harvested eggs were held in Petri dishes on moistened filter paper at 25°C, RH > 80%, in the dark. The Petri dishes were photographed and total eggs counted using the cell counter function of Image J software (Schneider et al., 2012). The number of larvae hatching from each dish was recorded daily until no further hatching was observed.

### qRT-PCR analysis

qRT-PCR analysis was performed in a similar manner as described previously [49]. The sequences for the forward and reverse primers used for the qRT-PCR are in S1 Table. The primers were validated using standard curves, based on serial dilutions of cDNA to determine the primer annealing efficiencies. A no-template control was included in each experiment to check for possible contamination. qRT-PCR (in technical duplicates) was performed using conditions that were previously described [49]. Relative quantification of the transcripts was calculated using the comparative 2^-δδCT^ method [50] and was normalized to *β-actin* [43, 51–53]. The specificity of qRT-PCR was confirmed by melting-curve analyses after each reaction.

### Statistical analysis

The means and standard errors of means (SEM) were analyzed by analysis of variance (ANOVA) (JMP 8; SAS Institute, Cary, NC), and means were separated using Tukey’s HSD test (*p* < 0.05) in manners similar to previous studies [49, 54]. For one-to-one comparisons, means and SEM were analyzed by ANOVA and means were separated by Student’s *t* test.

## Results

### Identification and annotation of WCR *Argonautes*

The transcriptomic hits of *Ago1*, *Ago2*, *Ago3*, and *Aubergine* were identified following iterative tBLASTn searches of a WCR transcriptome using *Drosophila* and *Tribolium* Argonaute protein sequences as queries (S1 Fig). The preliminary annotation of the WCR *Argonaute* transcripts was initially confirmed by the results of reciprocal BLASTp against databases from which query sequences were derived (data not shown).

**Fig 1:**
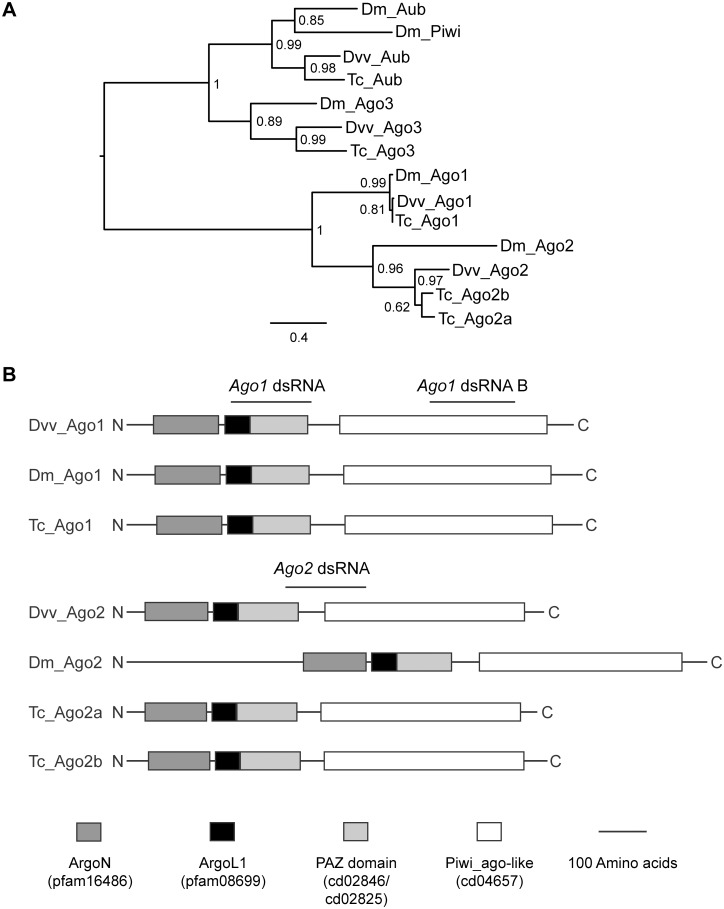
Phylogenetic tree and domain structure of Argonaute proteins of *Diabrotica virgifera virgifera* Le Conte and their homologs from selected species. (A) A phylogenetic analysis of Argonaute proteins from selected species. The amino acid sequences of the PIWI domains of Argonautes from *Diabrotica virgifera virgifera* (Dvv) were aligned with those of selected Argonautes from *D*. *melanogaster* (Dm) and *T*. *castaneum* (Tc). A midpoint-rooted tree was generated using a maximum likelihood approach (Guindon et al. 2010). aLRT SH-like branch support values are shown at the nodes. The scale bar represents the numbers of substitutions per site. (B) Schematic structures of Ago1 and Ago2 proteins from selected species. Names and Pfam IDs of conserved domains are shown in brackets.

The annotation of WCR *Argonautes* was further validated by phylogenetic and domain structure analyses (Fig 1). A maximum-likelihood phylogenetic tree, built based on the alignment of the conserved PIWI domain sequences of *Argonautes* from WCR, *T*. *castaneum* and *D*. *melanogaster*, shows that WCR *Argonautes* all clustered with their respective orthologs from *T*. *castaneum* and *D*. *melanogaster* (Fig 1A and S2 Fig). *Argonaute 1* from all species showed the highest degree of orthology among all *Argonaute* paralogs.

As shown in Fig 1B, WCR Ago1 and Ago2 share similar domain structure with their respective orthologs from *Drosophila* and *Tribolium*. They all possess ArgoN domains at their N termini, which are connected to the nucleic acid binding PAZ domains via linker domains (ArgoL1). Finally, they all have the canonical PIWI domains at their C termini.

We further evaluated the presence of conserved residues that are important for PIWI domain functions in WCR Argonaute proteins. Four residues (Tyr123, Lys127, Gln137 and Lys163) in the conserved motif (Tyr-Xaa3-Lys-Xaa(9–11)-Gln-Xaa(21–33)-Lys) of the PIWI domain that are believed to be involved in binding to the 5’ phosphate group of the first nucleotide of the guide RNA strand [55] are all present in WCR Argonautes (S2 Fig).

Also illustrated in S2 Fig, WCR Argonautes possess conserved metal chelating residues (Asp, Asp and His [DDH]) that are found in the catalytic motif of PIWI domain of other Argonaute proteins [18, 55, 56].

### Identification and annotation of WCR *Dicers* and *Drosha*

**Fig 2:**
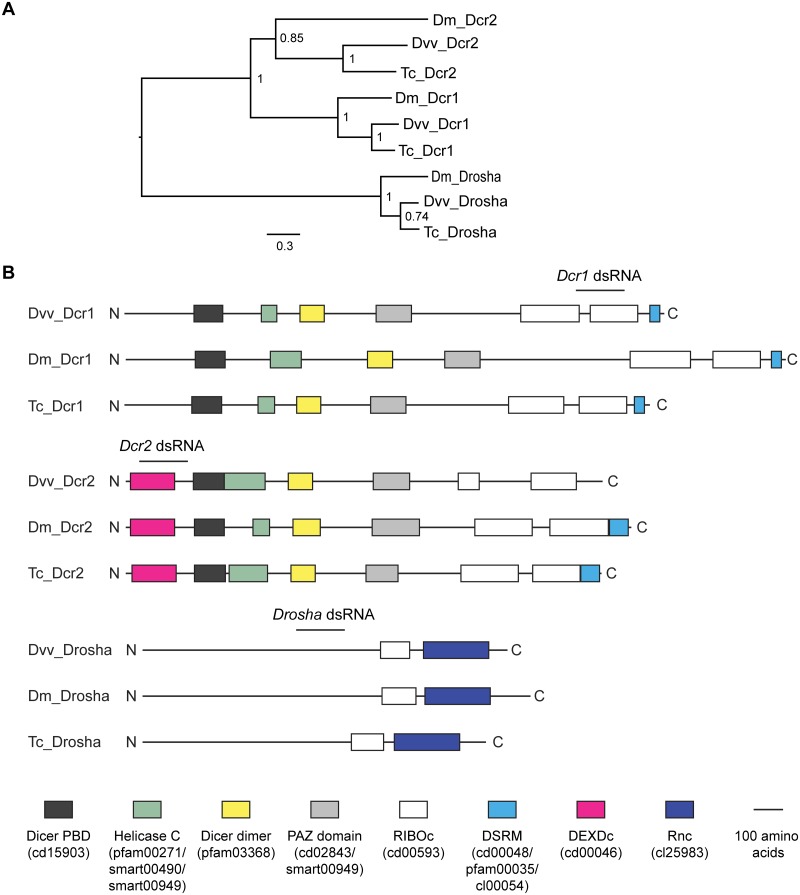
Phylogenetic tree and domain structure of Dicer and Drosha proteins of *Diabrotica virgifera virgifera* Le Conte and their homologs from selected species. (A) A phylogenetic analysis of Dicer and Drosha proteins from selected species. The full-length amino acid sequences of Dicers and Drosha from *Diabrotica virgifera virgifera* (Dvv) were aligned with those of selected Dicers and Droshas from *D*. *melanogaster* (Dm) and *T*. *castaneum* (Tc). A midpoint-rooted tree was generated using a maximum likelihood approach (Guindon et al. 2010). aLRT SH-like branch support values are shown at the nodes. The scale bar represents the numbers of substitutions per site. (B) Schematic structures of Dcr1, Dcr2, and Drosha proteins from selected species. Names and Pfam IDs of conserved domains are shown in brackets.

In addition to the previously identified *Dcr2*, we identified a *Dcr1* and a *Drosha* transcripts in the WCR transcriptome. A phylogenetic analysis shows that WCR *Dcr1*, *Dcr2*, and *Drosha* cluster with their respective orthologs from *Drosophila* and *Tribolium* (Fig 2A and S3 Fig). A domain structure comparison appears to support the phylogeny-based annotation. Dicer 1 proteins from all three insects have similar conserved domains: a dicer partner binding domain (PBD), a N-terminal helicase C domain, a dimerization domain, a PAZ domain, tandem RNase III domains (RIBOc), and a carboxyl dsRNA binding motif (DSRM) (Fig 2B). By comparison, Dcr2s from these insects have an additional N-terminal helicase domain (DEXDc). Interestingly, WCR Dcr2 differs from its homologs from *Drosophila* and *Tribolium* in that it lacks the C-terminal DSRM. Western corn rootworm Drosha is similar to orthologs from *Drosophila* and *Tribolium* in that they all contain a RIBOc domain and an Rnc domain that encodes for dsRNA-specific ribonuclease activity.

Results from previous studies of WCR Ago2 and Dcr2 suggest that these proteins are likely involved in the dsRNA pathway [42, 43]. Based on the results of the phylogenetic and domain structure analyses described above, we speculate that other WCR Argonautes, Dicer, and Drosha likely perform similar functions as their orthologs from *Drosophila*. Namely, Ago1, Dcr1, and Drosha are likely involved in the miRNA pathway; Ago3 and Aubergine are likely involved in the piRNA pathway.

### Loss-of-function analyses of *Ago1* and *Ago2*

Because we were interested in evaluating the possibility that WCR may develop resistance to dsRNA- or miRNA-based pest control measures through downregulating the expression of RNAi pathway components (*i*.*e*., *Ago1*, *Ago2*, *Dcr1*, *Dcr2*, and *Drosha*), we next performed loss-of-function analyses on these putative RNAi pathway genes using an RNAi-based approach.

**Fig 3:**
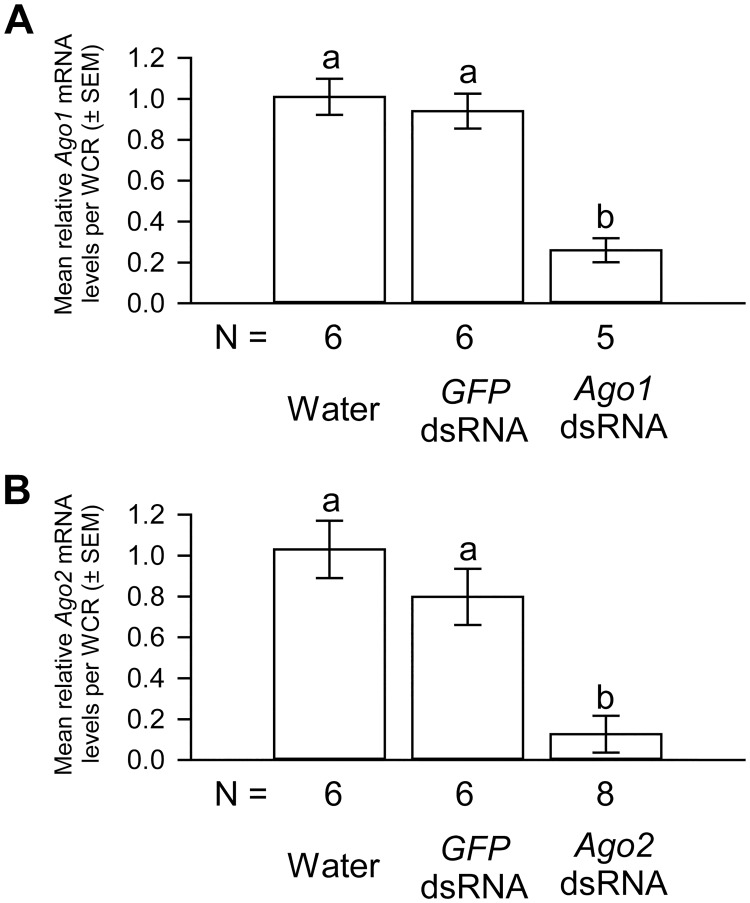
The effects of *Ago1* and *Ago2* dsRNA deliveries on the mRNA levels of *Ago1* and *Ago2*, respectively. *Ago1* (A) and *Ago2* (B) dsRNA deliveries in WCR females resulted in significant knockdown in the mRNA levels of *Ago1* and *Ago2*, respectively. One-way ANOVA results for the comparison of the relative *Ago1* (or *Ago2*) mRNA levels in WCR females that received various treatments are *F*_*2*,*16*_ = 24.2 for *Ago1* and *F*_*2*,*19*_ = 22.0 for *Ago2*, *P* < 0.000001 for both. The *Ago1* (or *Ago2*) mRNA levels in water control females were scaled to 1. Tukey-Kramer HSD lettering for all comparisons. Different letters denote significant differences. N represents the number of biological replicates.

When compared to water controls, oral delivery of *Ago1* and *Ago2* dsRNA resulted in a reduction of 74% and 83% in the *Ago1* and *Ago2* mRNA levels, respectively. *GFP* dsRNA treatment did not affect the levels of either *Ago1* or *Ago2* mRNA (Fig 3).

**Fig 4:**
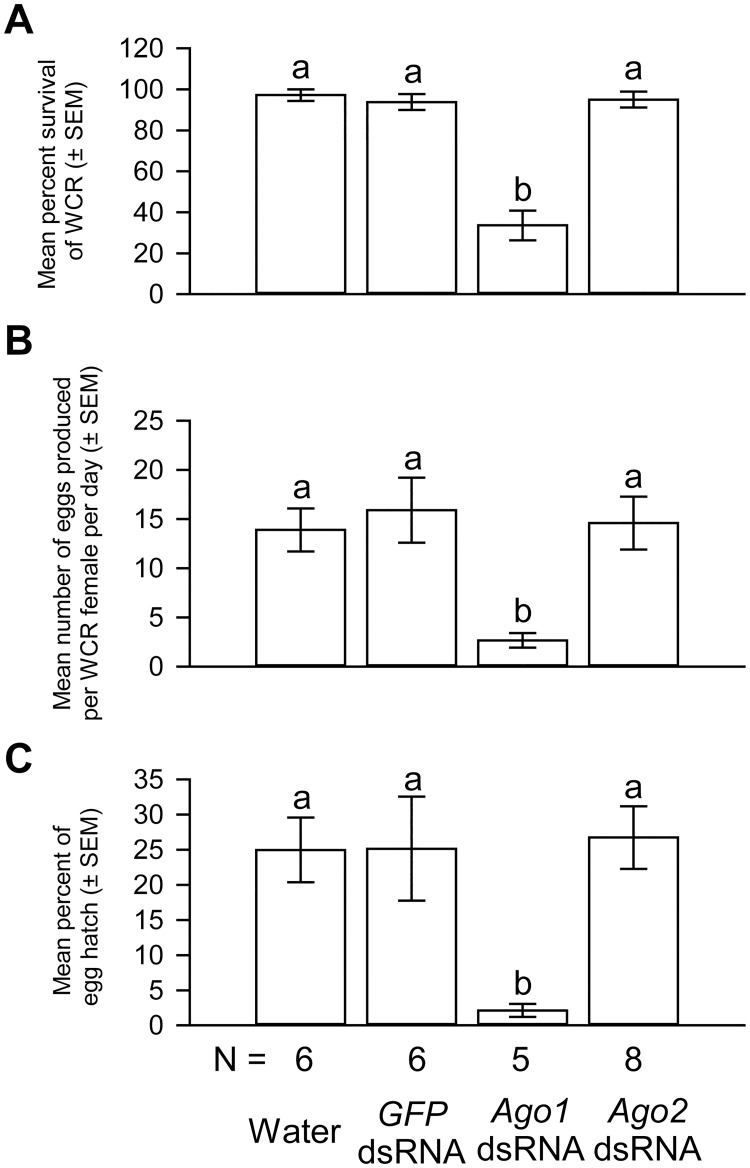
Functional effects of *Ago1* and *Ago2* dsRNA deliveries. *Ago1*, but not *Ago2*, dsRNA delivery significantly increased the mortality of WCR females (A), reduced the oviposition rate (B), and impaired the hatching of the few eggs produced by these females (C). One-way ANOVA: *F*_*3*,*24*_ = 45.5, *P* < 0.0001 for the comparison of survival rates; *F*_*3*,*24*_ = 4.7, *P* = 0.011 for the comparison of the oviposition rates; *F*_*3*,*24*_ = 4.5, *P* = 0.012 for the comparison of egg hatch rates. Tukey-Kramer HSD lettering for all comparisons. Different letters denote significant differences. N represents the number of biological replicates.

Almost all females treated with water, *GFP* or *Ago2* dsRNA survived after a 4-day oviposition period, whereas 64% of *Ago1* dsRNA-treated WCR females died during the same period (Fig 4A). Moreover, *Ago1* dsRNA treatment severely reduced the oviposition rate. Each WCR female treated with water produced, on average, 13.9 eggs per day (Fig 4B). By comparison, each WCR female treated with *Ago1* dsRNA produced only 2.6 eggs per day, representing a reduction of 81% relative to the water controls. In contrast, *Ago2* and *GFP* dsRNA-treated WCR females each produced 14.6 and 15.9 eggs per day, respectively, and these numbers were not significantly different from those of water controls.

The few eggs produced by *Ago1* dsRNA-treated females displayed reduced viability. Approximately 25% of the eggs produced by WCR females treated with water, *GFP* dsRNA or *Ago2* dsRNA hatched. In contrast, only 2.6% of the eggs produced by *Ago1* dsRNA-treated females hatched, representing a 92% reduction in egg hatch rate (Fig 4C).

The reduced viability of WCR females treated with *Ago1* dsRNA was somewhat unexpected because the loss of *Drosophila Ago1* function during the adult stage reduced the number of germline stem cells and impaired oogenesis, but had no effect on survival within the experiment period [31, 32]. Therefore, to confirm the effects we observed were specifically caused by *Ago1* knockdown, we performed a replicative experiment using a non-overlapping *Ago1* dsRNA (*Ago1* dsRNA B, position shown in Fig 1B) and a separate cohort of WCR. Only *GFP* dsRNA treatment was used as the negative control in this experiment because the effects induced by such treatment on either *Ago1* gene expression or fitness were indistinguishable from those caused by water treatment (Figs 3 and 4).

**Fig 5:**
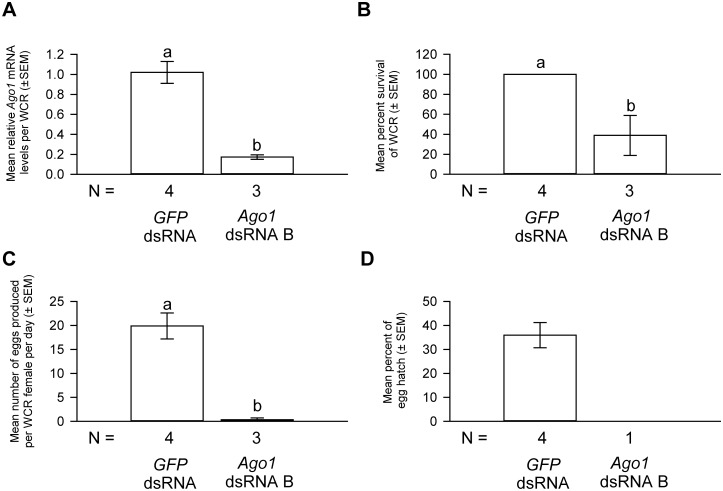
The effects of an alternative *Ago1* dsRNA delivery on fitness and *Ago1* gene expression in WCR females. *Ago1* dsRNA B delivery significantly reduced the *Ago1* mRNA levels in WCR females (A), increased the mortality of WCR females (B), reduced the oviposition rate (C), and impaired the hatching of the few eggs produced by these females (D). Student’s *t* test (2-tailed) results: *P* = 0.017 for the comparison of the relative *Ago1* mRNA levels; *P* = 0.015 for the comparison of survival rates; *P* = 0.0018 for the comparison of oviposition rates. No eggs deposited by *Ago1* dsRNA B-treated females hatched. Different letters denote significant differences. N represents the number of biological replicates.

As shown in Fig 5A, *Ago1* dsRNA B treatment resulted in an 83% reduction in the *Ago1* mRNA levels. Furthermore, *Ago1* dsRNA B treatment reduced the survival rate by approximately 60% (Fig 5B). As expected, the oviposition rate was also negatively affected by *Ago1* dsRNA B treatment. Each WCR female treated with *Ago1* dsRNA B deposited, on average, 0.37 egg per day. In contrast, *GFP* dsRNA-treated females laid approximately 20 eggs per day (Fig 5C). Finally, no larvae hatched from the few eggs deposited by *Ago1* dsRNA B-treated females (Fig 5D). Thus, the results from this replicative experiment are consistent with those from the experiment described above, suggesting that loss of *Ago1* function in WCR indeed negatively impacted adult survival, oviposition, as well as egg hatching.

Although no reduction in fitness parameters was observed in *Ago2* dsRNA-treated WCR females during a 4-day oviposition period, we could not rule out the possibility that the lack of effects on fitness could be due to the presence of residual Ago2 guide RNA complex that reportedly has a half-life on the order of days [57]. Therefore, we performed another experiment by exposing WCR females to continuous *Ago2* dsRNA treatment for a longer period of time and assessed the survival, oviposition and egg hatch rates for 4 weeks.

**Fig 6:**
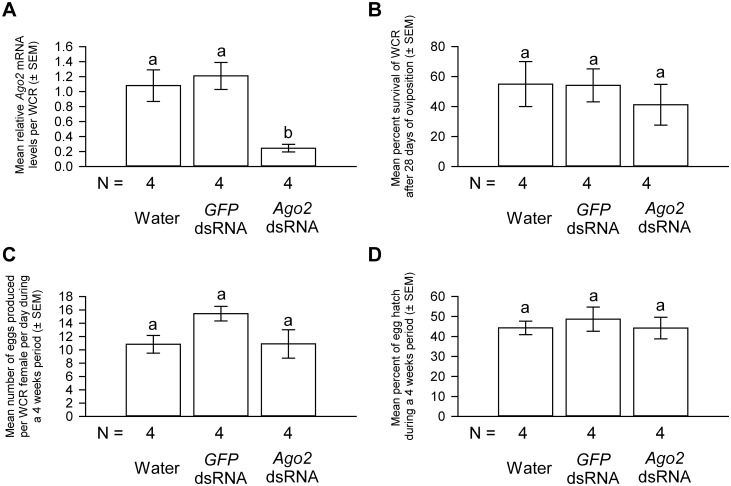
The effects of long-term *Ago2* dsRNA delivery on fitness and *Ago2* gene expression in WCR females. *Ago2* dsRNA delivery significantly reduced the *Ago2* mRNA levels (A), but did not affect the survival (B), oviposition (C) of WCR females or the hatch rate (D) of eggs produced by these females over a 28-days period. One-way ANOVA: *F*_*2*,*11*_ = 10.15, *P* = 0.0049 for the comparison of the relative *Ago2* mRNA levels; *F*_*2*,*11*_ = 0.33, *P* = 0.72 for the comparison of survival rates; *F*_*2*,*11*_ = 2.76, *P* = 0.11 for the comparison of oviposition rates; *F*_*2*,*11*_ = 0.25, *P* = 0.77 for the comparison of egg hatch rates. The *Ago2* mRNA levels in water control females were scaled to 1. Tukey-Kramer HSD lettering for all comparisons. N represents the number of biological replicates.

At the end of a 4-week oviposition period, the *Ago2* mRNA levels in *Ago2* dsRNA-treated WCR females were reduced by 75% when compared to water controls (Fig 6A). Approximately 55% and 41% of WCR in water control and *Ago2* dsRNA groups, respectively, survived during this period and the survival rates were not significantly different (Fig 6B). Females in water control and *Ago2* dsRNA groups each produced approximately 10 eggs per day (Fig 6C). Finally, approximately 44% of the eggs produced by both water and *Ago2* dsRNA groups hatched. Thus, long-term *Ago2* gene knockdown did not have any discernable effect on survival, oviposition, and egg hatching (Fig 6B–6D). Similar to the results from the short-term experiment, *GFP* dsRNA treatment had no effects on *Ago2* gene expression, survival, oviposition, and egg hatch rate when compared to water controls. Thus, the results from the short-term and long-term *Ago2* gene knockdown studies consistently showed that downregulation of *Ago2* expression did not impose a fitness cost to WCR females.

### Loss-of-function analyses of *Dcr1*, *Dcr2*, and *Drosha*

**Fig 7:**
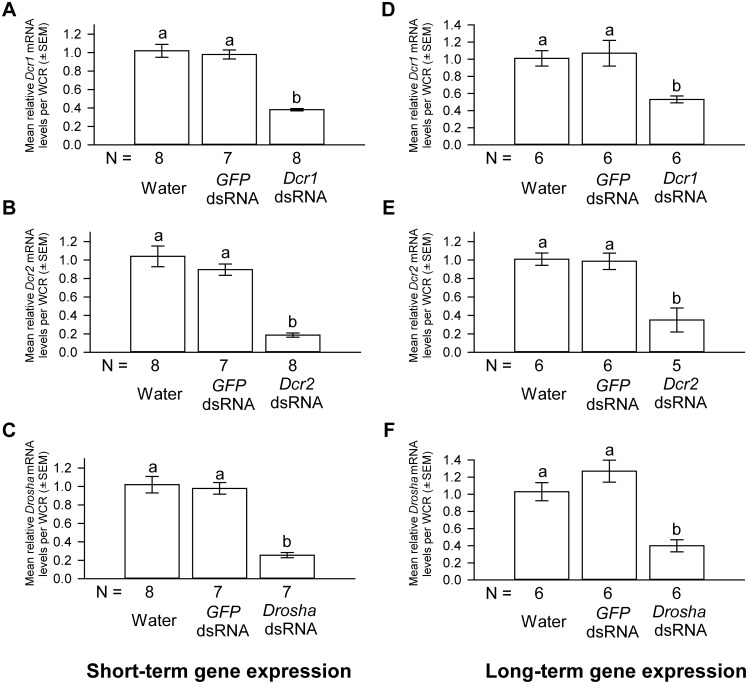
The effects of short-term and long-term *Dcr1*, *Dcr2*, *Drosha* dsRNA deliveries on the mRNA levels of *Dcr1*, *Dcr2*, and *Drosha*, respectively. Short-term *Dcr1* (A), *Dcr2* (B), and *Drosha* (C) dsRNA deliveries in WCR females resulted in significant knockdown in the mRNA levels of respective genes. For relative *Dcr1*, *Dcr2*, and *Drosha* mRNA level comparisons: one-way ANOVA, *F*_*2*,*22*_ = 52.70, *F*_*2*,*22*_ = 37.11, and *F*_*2*,*21*_ = 38.28 respectively, *P* < 0.0001 for all. Similarly, long-term *Dcr1* (D), *Dcr2* (E), and *Drosha* (F) dsRNA deliveries in WCR females resulted in significant knockdown in the mRNA levels of respective genes. For relative *Dcr1*, *Dcr2*, and *Drosha* mRNA level comparisons: one-way ANOVA, *F*_*2*,*17*_ = 8.12, *F*_*2*,*16*_ = 13.77, and *F*_*2*,*17*_ = 18.43 respectively, *P* < 0.005 for all. The *Dcr1*, *Dcr2*, and *Drosha* mRNA levels in water control females are scaled to 1. Tukey-Kramer HSD lettering for all comparisons. N represents the number of biological replicates.

**Fig 8:**
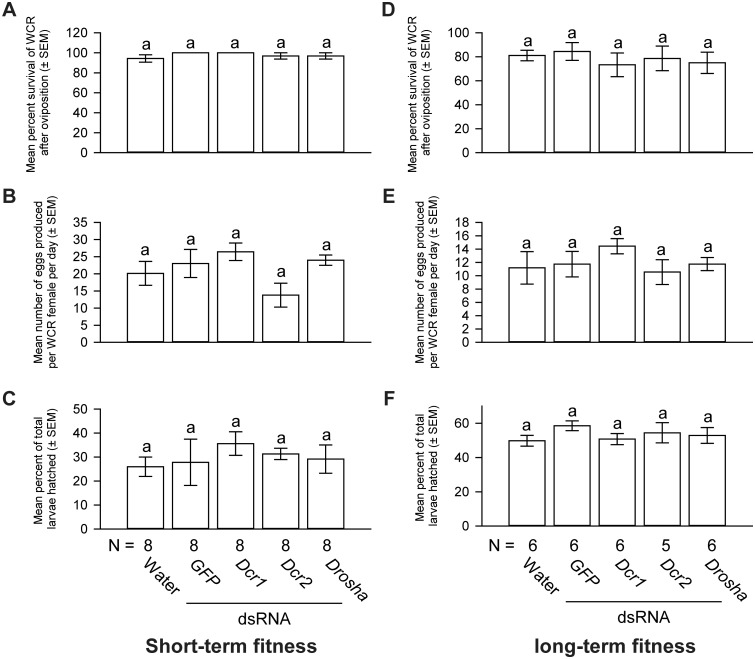
The effects of short-term and long-term *Dcr1*, *Dcr2*, and *Drosha* dsRNA deliveries on WCR fitness. Short-term *Dcr1*, *Dcr2*, and *Drosha* dsRNA deliveries had no effect on the survival of WCR females (A). These dsRNA deliveries did not affect the oviposition rates (B), or the egg hatch rates (C). One-way ANOVA: *F*_*4*,*39*_ = 0.86, *P* = 0.49 for the comparison of short-term survival rates; *F*_*4*,*39*_ = 2.33, *P* = 0.07 for the comparison of oviposition rates; *F*_*4*,*39*_ = 0.39, *P* = 0.81 for the comparison of egg hatch rates. Long-term *Dcr1*, *Dcr2*, and *Drosha* dsRNA deliveries had no effect on the survival (D) and oviposition rates (E) of WCR females, nor did they affect the hatch rates (F) of eggs produced by these females. One-way ANOVA: *F*_*4*,*28*_ = 0.30, *P* = 0.87 for the comparison of long-term survival rates; *F*_*4*,*28*_ = 0.71, *P* = 0.58 for the comparison of oviposition rates; *F*_*4*,*28*_ = 0.76, *P* = 0.55 for the comparison of egg hatch rates. Tukey-Kramer HSD lettering for all comparisons. N represents the number of biological replicates.

Short-term *Dcr1*, *Dcr2*, and *Drosha* dsRNA deliveries resulted in a reduction of 62%, 78% and 75% in *Dcr1*, *Dcr2*, and *Drosha* mRNA levels, respectively (Fig 7A–7C). Approximately 94–100% of WCR in each treatment group survived after a 4-day oviposition period and there was no significant difference in survivorship among treatment groups (Fig 8A). Western corn rootworm females in all treatment groups produced similar numbers of eggs (Fig 8B). Finally, similar percentages of eggs produced by WCR in different treatment groups hatched (Fig 8C).

To evaluate possible effects of long-term knockdown of *Dcr1*, *Dcr2*, and *Drosha* on fitness, we extended the fitness bioassays for another 24 days after the conclusion of the short-term experiment. As shown in Fig 7D–7F, a prolonged exposure to *Dcr1*, *Dcr2*, and *Drosha* dsRNAs resulted in a reduction of 47%, 65%, and 60% in *Dcr1*, *Dcr2*, and *Drosha* mRNA levels, respectively. Between 73% and 84% of WCR survived at the end of the long-term study and no significant difference in mortality was found between treatments (Fig 8D). As shown in Fig 8E, WCR females in each treatment group had similar oviposition rates of 10–14 eggs per female per day. The hatch rates of eggs produced by WCR that received one of the dsRNAs or control treatments were also not significantly different (Fig 8F).

## Discussion

In the current study, we identified five novel, putative members of the WCR RNAi pathway: *Ago1*, *Ago3*, *Aubergine*, *Dcr1*, and *Drosha*. Results from our phylogenetic analysis of *Argonautes* are consistent with those from a previous study of the evolution of these genes [24] that showed the phylogenetic tree of eukaryote *Argonautes* largely followed the phylogeny of these species. Taken together, these results suggest that the pathways (*i*.*e*., siRNA, miRNA, and piRNA) in which different Argonautes participate are likely conserved among these species. That the phylogenetic tree of *Dicers* and *Drosha* of these insects also follows the phylogeny of these species lends further support to the notion that these pathways involving small, non-coding RNAs are conserved across species.

Among all Argonautes, *Ago1s* show the highest degree of homology among different species. Similar observations have been made before. For example, studies of *Argonautes* from several vector mosquitos and drosophilid species showed that the *Ago1s* evolved at much lower rates than other Argonautes such as *Ago2s* [58, 59]. *Ago1* conservation implies an evolutionary trend of purifying selection, probably due to the conserved mechanisms in the biological pathways (*e*.*g*., development) involving this Argonaute protein. This hypothesis is supported by the conservation of many miRNAs among drosophilids, mosquitoes, and humans [60].

Results from our loss-of-function study of *Ago1* appear to support the idea that Ago1 plays essential roles in multiple biological pathways in WCR. Females with decreased *Ago1* expression suffered a clear reduction in fitness, such as increased mortality and reduced oviposition in those females that survived. Hatching of eggs produced by these females also decreased. Importantly, these defects were observed in two separate WCR cohorts treated with two non-overlapping *Ago1* dsRNAs (Figs 4 and 5). This suggests that these phenotypes resulted from specific knockdown of *Ago1* in WCR. That *Ago1* dsRNA treatment in mothers reduced the hatching of eggs they produced is consistent with the notion that RNAi response in WCR is systemic and parental [44, 52].

Our results draw parallels to those from previous studies of *Ago1* in other insects, which showed that loss of Ago1 function resulted in defects in oogenesis and embryogenesis [30–32, 61]. It is possible that *Ago1* knockdown resulted in similar defects in the ovaries and eggs of WCR. Taken together, these results suggest that Ago1 likely plays a conserved role in the physiological processes that are essential to oogenesis and embryogenesis in insects. Certainly, future studies are needed to investigate the precise causes for the reduced oviposition and egg hatching associated with *Ago1* gene knockdown in WCR.

Interestingly, there have been only a few studies investigating the effects of *Ago1* downregulation in adults, probably due to the fact that *Ago1* mutants are often embryonic lethal [32]. A study in *Drosophila* achieved *Ago1* downregulation during the adult stage specifically by withholding the daily heat shock that was needed for the expression of an exogenous *Ago1*, under the control of a heat shock promoter, in an *Ago1* mutant background. However, no increase in mortality was observed during the experimental period [32]. It is possible that the discrepancy in results between the current study and that of Yang et al. [32] is due to the difference in experimental approaches (*e*.*g*., presence and absence of previous heat shock treatment). It is also possible that the essential role that Ago1 plays in adult survival may be WCR-specific.

Results from previous studies suggest that Ago1 may play, in adults of other animals, important roles other than those found in sexual organs. Some studies showed that *Ago1* transcript was found in the head and the digestive systems of *Drosophila* adults (http://flybase.org). Another study found that human Ago1 bound directly to RNA polymerase II and the promoters of actively transcribed genes in cancer cells. Results from this latter study suggest that this protein may play a direct role in regulating the transcription of genes, some of which are important for cell growth and survival [62]. Taken together, these data suggest that Ago1 may play a diverse, or even critical, role in adults of other animals as well.

Further studies are needed to reveal the details of pathways (*e*.*g*., miRNA) that WCR Ago1 is involved in. Similar to dsRNA-based approaches, miRNA-based measures also have the potential to be effective in controlling pest insects [38]. If Ago1 is indeed critical to the miRNA pathway in WCR, then it is unlikely that this pest would develop resistance to miRNA-based pest control measures by downregulating the expression of *Ago1*, due to the high fitness cost associated with such action.

In contrast to the severe effects that decreased *Ago1* expression had on the fitness of WCR adults, reduced expression of *Ago2* and *Dcr2* genes had no detectable impact on the viability and oviposition rate of WCR females. Nor did it affect the egg hatching. These results suggest that these genes may not be critical to the survival, oogenesis, and embryonic development of WCR.

It is possible that reduced *Dcr2* and *Ago2* expression may still negatively affect the fitness of WCR because both have been shown to participate in antiviral defense in other insects [63–66]. It is possible that reduced *Dcr2* or *Ago2* expression in WCR may impair its ability to resist viral infection and may result in reduced fitness, if such infection is virulent. Aphid lethal paralysis virus and an Iflavirus have been found in WCR from field collections in the U.S. and Europe [67, 68]. It is unclear how prevalent or virulent these viral infections are and future studies are needed to elucidate whether downregulation of *Ago2* or *Dcr2* affects the ability of WCR to defend against infections such as those caused by these viruses.

Our results have potential implications for pest management strategies that rely on the dsRNA gene silencing pathway and suggest that WCR may develop resistance against transgenic plants expressing WCR-specific dsRNA by downregulating the expression of *Ago2* or *Dcr2*, especially if such downregulation does not affect WCR’s ability to defend against potential virulent viral infection.

The lack of effects on fitness of WCR females that received *Dcr1* or *Drosha* dsRNAs was somewhat unexpected, considering that a previous study showed ablation of *Ago1* and its miRNA biogenesis partners, *Dcr1* and *Drosha*, all reduced oocyte formation and germline cell division in *Drosophila* [31]. It is important to note that the exact roles that Ago1, Dcr1, and Drosha proteins play in the miRNA pathway in WCR remain unknown. Assuming that these proteins are all involved in the miRNA pathway in WCR, several possibilities may account for the discrepancy in the fitness phenotypes seen in WCR treated with *Ago1*, *Dcr1*, and *Drosha* dsRNAs. First, it is possible that *Dcr1* and *Drosha* expression was not sufficiently reduced to alter the fitness phenotypes. An alternative *Dcr1* dsRNA construct was initially tested and found to produce similar knockdown rates as the construct used in the current study (data not shown). Thus, it is possible that WCR *Dcr1* may be difficult to knock down strongly using an RNAi-based method. If so, alternative approaches (*e*.*g*., genomic editing) may be needed for a definitive loss-of-function study of *Dcr1*, and possibly *Drosha*. Secondly, it is possible the effects on fitness induced by the downregulation of *Dcr1* and *Drosha* may manifest beyond our experiment time period. However, this possibility appears unlikely, considering that the half-lives of Dicer and Drosha (of human) are both on the order of hours [69, 70]. At the end of our long-term experiments, the mean age of WCR was 46 days (18 days of pre-oviposition + 28 days of oviposition). WCR reared under laboratory conditions can live up to 90 days [71]. If WCR Dcr1 and Drosha proteins are extremely stable, it is possible that a more extended study (*e*.*g*., covering the whole life span of WCR) may be needed to evaluate the effects on fitness caused by the knockdown of *Dcr1* or *Drosha*.

It is also possible that other proteins, such as Dcr2, may be involved in miRNA biogenesis in WCR. Dicer 2 has been shown to play a critical role in the dsRNA pathway in WCR and is postulated to be involved in the biogenesis of siRNA [42, 43]. However, it is unclear whether Dcr2 plays a role in the miRNA pathway in WCR. Interestingly, some insects (*e*.*g*., *Aleochara curtula*, a beetle) apparently lack *Dcr1*, but possess *Dcr2* [25]. It is possible that Dcr2 protein in these insects (and possibly other insects as well) may possess the ability to process miRNAs. If Dcr2 is involved in the processing of miRNAs in WCR, then knockdown of both *Dcr1* and *Dcr2* may be needed to completely impair the biogenesis of miRNAs. Finally, it is possible that our previous assumption may be incorrect and that Dcr1 and Drosha may be involved in pathways other than that participated by Ago1 in WCR. Therefore, knockdown of neither *Dcr1* nor *Drosha* can phenocopy *Ago1* knockdown.

In summary, we showed that downregulation of *Ago1*, *Ago2*, *Dcr1*, *Dcr2*, and *Drosha* in WCR adults resulted in different fitness phenotypes. These results suggest that they likely play distinct physiological roles during the adult stage of this corn pest. Of the five genes examined in this study, only the knockdown of *Ago1* resulted in adult mortality and reproductive defects, suggesting that this protein plays essential roles in multiple physiological pathways in WCR. The observations that *Dcr1* and *Drosha* knockdowns in WCR did not phenocopy *Ago1* knockdown raise the possibility that the roles these genes play may be different from those of *Drosophila* orthologs. Further studies are needed to reveal the pathways these genes are involved in and to investigate whether knockdown of these genes affect fitness in other life stages (e.g. larva).

## Supporting information

S1 Table Sequences of PCR primers used in the current study. (DOCX)

S1 Fig The nucleotide and deduced amino acid sequences of WCR *Ago1*, *Ago2*, *Dcr1*, *Dcr2*, and *Drosha* transcripts. (TXT)

S2 Fig A multiple sequence alignment of the PIWI domains of Argonaute proteins from WCR and select species. (DOCX)

S3 Fig A multiple sequence alignment of the amino acid sequences of Dicers and Drosha from WCR and select species. (DOCX)
