# Supplementary material for: Distinct fitness costs associated with the knockdown of RNAi pathway genes in western corn rootworm adults
Source: PLoS One. 2017 Dec 21;12(12):e0190208. doi: 10.1371/journal.pone.0190208 (PMC5739497; doi:10.1371/journal.pone.0190208)
Supplement: S1 Table — (DOCX) [file pone.0190208.s001.docx]

**S1 Table**. Sequences of PCR primers used in the current study.

| Gene | Primer sequence |
| --- | --- |
|  | dsRNA synthesis* |
| *Ago1* | DvvAgo1F  5’-TAATACGACTCACTATAGGGAGGGTGGTAGAGAAGTCTGGTTTG-3’  DvvAgo1R  5’-TAATACGACTCACTATAGGGAGCGAAGTCTGCATGTCCGTTA-3’  DvvAgo1bF  5’-TAATACGACTCACTATAGGGAGAGAGTTGGCGATACTGTGTTAG-3’  DvvAgo1bR  5’-TAATACGACTCACTATAGGGAGCCTCTTCTGGACCACGATAAAT-3’ |
| *Ago2* | DvvAgo2F  5’-TAATACGACTCACTATAGGGAGTATCCTCAGATGCCGACACTA-3’  DvvAgo2R  5’-TAATACGACTCACTATAGGGAGGGTTGTTCTGCTTCACCAATC-3’ |
| *Dcr1* | DvvDcr1F  5’-TAATACGACTCACTATAGGGAG AGGCTACCAGATGATGGTTATG-3’  DvvDcr1R  5’-TAATACGACTCACTATAGGGAG TTTCCTCACATTCGGTCTCTAC-3’ |
| *Dcr2* | DvvDcr2F  5’-TAATACGACTCACTATAGGGAGCATACAGTGAGGGCGGTAAA-3’  DvvDcr2R  5’-TAATACGACTCACTATAGGGAGTTCCTGAGGGTTTGTGGAATAG-3’ |
| *Drosha* | DvvDroF  5’- TAATACGACTCACTATAGGGAGTGCTCCCAGTTCCATTTCC-3’  DvvDroR  5’- TAATACGACTCACTATAGGGAGCACAGTCACGTCTCTCTTCATC-3’ |
| *GFP* | GfpF  5’-TAATACGACTCACTATAGGGAG AGGTGATGCTACATACGGAAAG-3’  GfpR  5’-TAATACGACTCACTATAGGGAG ACAGGTAATGGTTGTCTGGTAAA-3’ |
|  | Quantitative reverse transcriptase PCR |
| *Ago1* | rtDvvAgo1F  5’-AAGTCCACCTTCCAGTCTTTG-3’  rtDvvAgo1R  5’-TGGCCATTCCTAACACAGTATC-3’ |
| *Ago2* | rtDvvAgo2F  5’-CCGACGTACTATGCCCATTTAG-3’  rtDvvAgo2R  5’-CTTCTGGATACTGTCCTGGATTT-3’ |
| *Dcr1* | rtDvvDcr1F  5’-GTTGCTGAGGCTCTCAGATTAG-3’  rtDvvDcr1R  5’-CTCTTCCTCCCTGGATTTCTTG-3’ |
| *Dcr2* | rtDvvDcr2F  5’-AGTTCAACCAGACGAGAAAGG-3’  rtDvvDcr2R  5’-GGTTTCCAGACGTTCCAGATTA-3’ |
| *Drosha* | rtDvvDroF3  5’-TGCGTGAGCTGGAATTGT-3’  rtDvvDroR3  5’-GAATCTCGGCAGGAAATGGA-3’ |
| *Actin* | rtDvvActinF  5’-TCCAGGCTGTACTCTCCTTG-3’  rtDvvActinR  5’-CAAGTCCAAACGAAGGATTG-3’ |

*T7 polymerase promoter sequence (**TAATACGACTCACTATAGGGAG**) was added to 5’ end of the oligonucleotide sequence to facilitate subsequent dsRNA transcription as described in manufacturer’s protocol (MEGAScript, Ambion).
