# Supplementary material for: Distinct fitness costs associated with the knockdown of RNAi pathway genes in western corn rootworm adults
Source: PLoS One. 2017 Dec 21;12(12):e0190208. doi: 10.1371/journal.pone.0190208 (PMC5739497; doi:10.1371/journal.pone.0190208)
Supplement: S2 Fig — (DOCX) [file pone.0190208.s003.docx]

**S2 Fig.**

A multiple sequence alignment of the PIWI domains of Argonaute proteins from *Diabrotica virgifera virgifera* Le Conte (Dvv) and select species. Four conserved residues involved in binding of guide RNA are highlighted in bold. Three conserved residues involved in slicing activity are in bold and italicized. Argonaute protein sequences were retrieve from GenBank. The accession numbers for *Drosophila melanogaster* (Dm) Ago1, Ago2, Ago3, Aubergine and Piwi are NP_725341.1, NP_648775.1, ABO27430.1, NP_476734.1 and NP_476875.1, respectively. The accession numbers for *Tribolium castaneum* (Tc) Ago1, Ago2a, Ago2b, Ago3 and Aubergine are XP_008196655.1, NP_001107842.1, NP_001107828.1, XP_968053.2 and XP_008196303.1, respectively.

Tc_Ago2a HDLIIVVVPN-SGPQ**Y**SLV**K**QAAELNVGCLT**Q**CIKERTIA--KLNP----QIIANILLKI

Tc_Ago2b YDLIIVVVPN-SGPQ**Y**SFV**K**QAAELNVGCLT**Q**CIKERTIG--RLNP----QTVGNILLKI

Dvv_Ago2 YDVIFVVVPN-SGPQ**Y**SYV**K**TAAEINVGCLT**Q**CVKVRTVL--KMNS----QTALNLLLKV

Dm_Ago2 HDLAIVIIPQ-FRIS**Y**DTI**K**QKAELQHGILT**Q**CIKQFTVE-RKCNN----QTIGNILLKI

Tc_Ago1 LQLVVVVLPG-KTPV**Y**AEV**K**RVGDTVLGMAT**Q**CVQAKNVN--KTSP----QTLSNLCLKI

Dvv_Ago1 LQLVVVVLPG-KTPV**Y**AEV**K**RVGDTVLGMAT**Q**CVQAKNVN--KTSP----QTLSNLCLKI

Dm_Ago1 LQLVVVVLPG-KTPV**Y**AEV**K**RVGDTVLGMAT**Q**CVQAKNVN--KTSP----QTLSNLCLKI

Tc_Ago3 TQVAVFICPTLRADR**Y**SII**K**KMCCVNIPVAS**Q**VILSKTL----SNPQKVRTIIHKIAMQI

Dvv_Ago3 NQIVVFICPTMRSDR**Y**GHI**K**KMCCSQIPVPS**Q**VINSRTL----SNPGKVRSIVQKIALQM

Dm_Ago3 TQMVVCICHNRRDDR**Y**AAI**K**KICCSEIPIPS**Q**VINAKTL----QNDLKIRSVVQKIVLQM

Tc_Aub PTMILVILPNNSTER**Y**SAI**K**KKCYVDRGIPT**Q**MFVARNL----TSK-GVMSIATKVAIQM

Dvv_Aub PTLIMCVLPTNQADR**Y**GSI**K**KKCYVDRGIPS**Q**IVLRKNL----TSK-GVMSIATKVAIQL

Dm_Aub PQIVMVVMRSPNEEK**Y**SCI**K**KRTCVDRPVPS**Q**VVTLKVIAPRQQKPTGLMSIATKVVIQM

Dm_Piwi PKLILCLVPNDNAER**Y**SSI**K**KRGYVDRAVPT**Q**VVTLKTT----KKPYSLMSIATKIAIQL

: . : * :* :* . . :: :::

Tc_Ago2a NS**K**LNGTNHILS--SRLPIMSRPCIIMGA***D***VTHPGPDAKDVPSVAAVTASHDPNAF-QYN

Tc_Ago2b NS**K**MNGTNHRLSPNSRPLIMKRPCMIMGA***D***VTHPSPDARDIPSVAAVTASHDPNAF-QYN

Dvv_Ago2 NA**K**LNGTNHFLS--TRPPILNRPTMIMGA***D***VTHPSPDSQHIPSVAAVTASYDPKAF-KYN

Dm_Ago2 NS**K**LNGINHKIKDDPRL-PMMKNTMYIGA***D***VTHPSPDQREIPSVVGVAASHDPYGA-SYN

Tc_Ago1 NV**K**LGGINSILVPSIRPKIFNEPVIFLGA***D***VTHPPAGDNKKPSIAAVVGSMDAHPS-RYA

Dvv_Ago1 NV**K**LGGINSILVPSIRPKIFNEPVIFLGA***D***VTHPPAGDNKKPSIAAVVGSMDAHPS-RYA

Dm_Ago1 NV**K**LGGINSILVPSIRPKVFNEPVIFLGA***D***VTHPPAGDNKKPSIAAVVGSMDAHPS-RYA

Tc_Ago3 TC**K**LGGTLWSVK------IPVSGWMVCGI***D***VYHGANNQ----SVCGFVASINGSMT-KYF

Dvv_Ago3 TC**K**LGGTLWTVR------FPFKGWMICGI***D***VYHGKKSS----SVCGFVSSLNESVS-RWF

Dm_Ago3 NC**K**LGGSLWTVK------IPFKNVMICGI***D***SYHDPSNRGN--SVAAFVASINSSYS-QWY

Tc_Aub NC**K**IGGAPWCVP------IPLSGLMVVGY***D***VCRDTVNKKK--SFAGIVGSLDKNIS-RFY

Dvv_Aub NC**K**IGGAPWSIV------MPLSNVMVVGY***D***VCRDTLKKQS--SFAAMVATLDRAMT-RYY

Dm_Aub NA**K**LMGAPWQVV------IPLHGLMTVGF***D***VCHSPKNKNK--SYGAFVATMDQKESFRYF

Dm_Piwi NC**K**LGYTPWMIE------LPLSGLMTIGF***D***IAKSTRDRKR--AYGALIASMDLQQNSTYF

. *: : : * * : : .. .: : :

Tc_Ago2a ICWRLQPPKVEIIEDLCAITVEQLMFFYRKT-RHKPETIVFFR***D***GVSEGQFAEVRRAEIS

Tc_Ago2b ICWRLQPPKVEIIEDLCNITVEQLKFFYQKT-GFKPESIVFFR***D***GVSEGQFKQVQRAEIA

Dvv_Ago2 ICWRLQPPRQEIIEDLENIVVDQLKFFYESNKGQKPQRIIFFR***D***GVSDGQFEQVTSAEVR

Dm_Ago2 MQYRLQRGALEEIEDMFSITLEHLRVYKEYR-NAYPDHIIYYR***D***GVSDGQFPKIKNEELR

Tc_Ago1 ATVRVQQHRQEIIQELSSMVRELLIMFYKSTGGYKPHRIILYR***D***GVSEGQFLQLLQHELT

Dvv_Ago1 ATVRVQQHRQEIIQELSSMVRELLIMFYKSTGGYKPHRIILYR***D***GVSEGQFLQLLQHELT

Dm_Ago1 ATVRVQQHRQEIIQELSSMVRELLIMFYKSTGGYKPHRIILYR***D***GVSEGQFPHVLQHELT

Tc_Ago3 SKAMFQDG--EIGDYFKMPFRQMLQAAKDRE-GAFPSKVIVFR***D***GVGDGQLEHCRKYEIT

Dvv_Ago3 STATFQAG--ELGDFMKTAFIKALEATRNAN-GYYPKKVVIVR***D***GVGDGQLTHCQRYEVK

Dm_Ago3 SKAVVQTKREEIVNGLSASFEIALKMYRKRN-GKLPTNIIIYR***D***GIGDGQLYTCLNYEIP

Tc_Aub NICCEHKMEEELSDNFAAAVVLLCKQYKEQN-GHYPERILIYR***D***GVGEGQLPFVVEHEVA

Dvv_Aub NHVTEHQLQEELSENISAFLIVACQKYKEVN-GRYPERILIYR***D***GVGDGQLPFVFTMEVE

Dm_Aub STVNEHIKGQELSEQMSVNMACALRSYQEQH-RSLPERILFFR***D***GVGDGQLYQVVNSEVN

Dm_Piwi STVTECSAFDVLANTLWPMIAKALRQYQHEH-RKLPSRIVFYR***D***GVSSGSLKQLFEFEVK

: : * :: ***:..*.: *:

Tc_Ago2a AIHQACKKLQREGY----EPRITFLVVQKRHHTRLFPTNPRDSEDRNNNVPAGTCVDTHI

Tc_Ago2b AIQKACKMLQKDDY----EPKITFLVVQKRHHTRLFPTNPRDSEDKNNNVPAGTCVDTHI

Dvv_Ago2 AIRAACKRVQREGY----EPAITFLVVQKRHHTRLFPLNPRDSHDRNLNVPAGTCVDTHI

Dm_Ago2 CIKQACDKV---GC----KPKICCVIVVKRHHTRFFPSGDVTTSNKFNNVDPGTVVDRTI

Tc_Ago1 AIREACIKLES-DY----KPGITFIVVQKRHHTRLFCADKKEQSGKSGNIPAGTTVDVGI

Dvv_Ago1 AIREACIKLEA-DY----KPGITFIVVQKRHHTRLFCSDKKEQSGKSGNIPAGTTVDVGI

Dm_Ago1 AIREACIKLEP-EY----RPGITFIVVQKRHHTRLFCAEKKEQSGKSGNIPAGTTVDVGI

Tc_Ago3 QLQEVIKEL---NI----ETTITFVVVQKRINTRIF---RTVNETNFENPPSGTVVDNMV

Dvv_Ago3 QLESCLKEL---EL----DIKICFVVVQKRISTRIF----SQGHDGADNPPCGTILDHSI

Dm_Ago3 QFEMVCGN----------RIKISYIVVQKRINTRIF----SGSGIHLENPLPGTVVDQHI

Tc_Aub NIKRKLQEEIYING----EVKMAFVVVSKRINTRIF--------TEKDNPPPGTVVDDVI

Dvv_Aub RIKEKLTSEIYKNG----DLKMSFIVVSKRINTRFF--------TPNSNPPPGTVVDDIV

Dm_Aub TLKDRLDEI-YKSAGKQEGCRMTFIIVSKRINSRYF--------TGHRNPVPGTVVDDVI

Dm_Piwi DIIEKLKTE-YARV-QLSPPQLAYIVVTRSMNTRFF--------LNGQNPPPGTIVDDVI

: : ::* : :* * * ** :* :

Tc_Ago2a TNPMMQDFYLVSHASIQGVAKPTKYCTLWDDNNMSNDDIEELTYYLCHMFTRCNRSVSYP

Tc_Ago2b TNPRMQDFYLVSHASIQGVAKPTKYCTLWDDNNMNNDDIEELTYHLCHMFTRCNRSVSYP

Dvv_Ago2 THPFMQDFYLVSHASIQGVAKPTKYCTLWDDNDMSNDDIEQLTYFLCHMFTRCNRSVSYP

Dm_Ago2 VHPNEMQFFMVSHQAIQGTAKPTRYNVIENTGNLDIDLLQQLTYNLCHMFPRCNRSVSYP

Tc_Ago1 THPTEFDFYLCSHQGIQGTSRPSHYHVLWDDSHLDSDELQCLTYQLCHTYVRCTRSVSIP

Dvv_Ago1 THPTEFDFYLCSHQGIQGTSRPSHYHVLWDDSHLDSDELQCLTYQLCHTYVRCTRSVSIP

Dm_Ago1 THPTEFDFYLCSHQGIQGTSRPSHYHVLWDDNHFDSDELQCLTYQLCHTYVRCTRSVSIP

Tc_Ago3 TRRQFYDFFLVPQSVRQGTVNPTHYVVLVDEGNIKPDHLQRLAYKLCHLYYNWSGTIRVP

Dvv_Ago3 TRRQLPDFFLVPQSVRQGTVNPTHYIVLHDTCGLKPDHIQRLCYKLCHLYYNWPGTIRVP

Dm_Ago3 TKSNMYDFFLVSQLVRQGTVTPTHYVVLRDDCNYGPDIIQKLSYKLCFLYYNWAGTVRIP

Tc_Aub TLPERYDFYIVSQCVRQGTVAPTSYNVIEDSMGLPPEKLQYLTYKLTHMYYNWSGTVRVP

Dvv_Aub TLPERFDFFVVSQCVNQGTVAPTSYNVLSDNMGLSPDRLQMLTYKLCHMYYNWSGTVRVP

Dm_Aub TLPERYDFFLVSQAVRIGTVSPTSYNVISDNMGLNADKLQMLSYKMTHMYYNYSGTIRVP

Dm_Piwi TLPERYDFYLVSQQVRQGTVSPTSYNVLYSSMGLSPEKMQKLTYKMCHLYYNWSGTTRVP

. :*:: .: *. *: * .: . : :: * * : . : . : *

Tc_Ago2a APTYYA***H***LAAARAKVYVE

Tc_Ago2b APTYYA***H***LAAARAKVYIE

Dvv_Ago2 APTYYA***H***LAAARGKVYIE

Dm_Ago2 APAYLA***H***LVAARGRVYLT

Tc_Ago1 APAYYA***H***LVAFRARYHLV

Dvv_Ago1 APAYYA***H***LVAFRARYHLV

Dm_Ago1 APAYYA***H***LVAFRARYHLV

Tc_Ago3 APCLYA***H***KLAAIVGQYIK

Dvv_Ago3 APCMYA***H***KLASLVGQHLK

Dm_Ago3 ACCMYA***H***KLAYLIGQSIQ

Tc_Aub APCQYA***H***KLAFMVSQYIH

Dvv_Aub APCQYA***H***KLAFMTAQHLH

Dm_Aub AVCHYA***H***KLAFLVAESIN

Dm_Piwi AVCQYA***K***KLATLVGTNLH

* *: * :
