# Supplementary material for: Distinct fitness costs associated with the knockdown of RNAi pathway genes in western corn rootworm adults
Source: PLoS One. 2017 Dec 21;12(12):e0190208. doi: 10.1371/journal.pone.0190208 (PMC5739497; doi:10.1371/journal.pone.0190208)
Supplement: S3 Fig — (DOCX) [file pone.0190208.s004.docx]

**S3 Fig.**

A multiple sequence alignment of the amino acid sequences of Dicers and Drosha from *Diabrotica virgifera virgifera* Le Conte (Dvv) and select species. Dicer protein sequences were retrieve from GenBank. The accession numbers for *Drosophila melanogaster* (Dm) Dicer 1, Dicer2, and Drosha are NP_524453.1, NP_523778.2, and NP_477436.1, respectively. The accession numbers for *Tribolium castaneum* (Tc) Dicer 1, Dicer2, and Drosha are XP_008199045.1 and NP_001107840.1, and XP_008199088.1, respectively.

Tc_Dcr2 ----------MDEEDELKPRNYQVNLMEIAIRENTIIYLPTGSGKTFIAIMVLKQLCAPI

Dvv_Dcr2 ----------MSSQD-LIPRNYQVLLMKICLEQNTIIYLPTGSGKTFITTMVLKQKGEDL

Dm_Dcr2 ----------MEDVE-IKPRGYQLRLVDHLTKSNGIVYLPTGSGKTFVAILVLKRFSQDF

Tc_Dcr1 MAC--YLNENVYTHT-FTPREYQVELLDSAKKRNTIVCSSASSAKAFITIKLLQEFSHKM

Dvv_Dcr1 MAS--YHNENVYTHT-FTPKEYQVELLESAKIKNTIMCSSTSCAKAFILVKLLQEFSWQM

Dm_Dcr1 MAFH-WCDNNLHTTV-FTPRDFQVELLATAYERNTIICLGHRSSKEFIALKLLQELSRRA

Dm_Drosha -----MYQPPLP------PPPVQPAPPPPPPPPEEDLSPP--------------------

Tc_Drosha MSDPYYYNSSYGQYV-NYPPNY---------PS---------------------------

Dvv_Drosha MGDHQWYYDNL-----NYPPPTQAQYNPHIPPQ---------------------------

*

Tc_Dcr2 LRPYSDGGKISVILVN--SVALVDQHGKYVRDHATFSVGTYTGEMNVDFWSEAE-----W

Dvv_Dcr2 LKSYSEGGKISIILVN--TVALVDQHGSYITNHTSFSVGKYTGEMNLDFWPRTK-----W

Dm_Dcr2 DKPIESGGKRALFMCN--TVELARQQAMAVRRCTNFKVGFYVGEQGVDDWTRGM-----W

Tc_Dcr1 RV---PHGKQALFVLDGPNVPIMTSHVKLLTDLTVTSI---------DKEENPP------

Dvv_Dcr1 RI---KNGKKALFILDPQNVPIMTSHIKYLTDLNCISIMEYT----LDAKENGI------

Dm_Dcr1 R----RHGRVSVYLSC--EVGTSTEPCSIYTMLTHLT--------DLRVWQEQPDMQIPF

Dm_Drosha ------------------GVGVPSHN---------------------------------Y

Tc_Drosha ----------------------PDPR---------------------------------W

Dvv_Drosha ----------------------SSHS---------------------------------Y

Tc_Dcr2 EQQFNKYQVVIMTSQIMVNLINNRFID-LGKVNLMIFDECHHGVEDQPMRQIMKHF---H

Dvv_Dcr2 FNEFNQYQVLIMTSQILDNLSRTDYID-LNKVNLLVFDECHRGVNDHTMRNLMKRF---E

Dm_Dcr2 SDEIKKNQVLVGTAQVFLDMVTQTYVA-LSSLSVVIIDECHHGTGHHPFREFMRLF-TIA

Tc_Dcr1 --SLKASNVIVTTAEVCVLLCKKNFVH-LDSYALIVI-DCLYGGQQSLVREIMARY--QA

Dvv_Dcr1 LDYFEPYQVITSTAEICVEMLKQSIISDFSAFNLFVIDDCLYGPRQSLIEIIMKYY-RSS

Dm_Dcr1 DHCWTDYHVSILRPEGFLYLLETRELL-LSSVELIVLEDCHDSAVYQRIRPLFENHIMPA

Dm_Drosha SSNESHSQ--------------------SSKSLDYVYPE----TPAPYASSVPSYDPYQQ

Tc_Drosha -PSWQWNQ--------------------Q------PYP------------PMMPPYPPPA

Dvv_Drosha -TQWSQSQ--------------------QTSTNVYSYP------------PVPSYPPPPI

: .

Tc_Dcr2 SCTDKPRVLGLTATLLNGNCKLSKVMDEIRSLEVTFHSKVATVE---GLDVVVGYSTNPQ

Dvv_Dcr2 HLIDPPRVIGLTATLLNGNCQPHEVLPKIRSLETTFHSKVATVE---ELKDVIGYSTNPQ

Dm_Dcr2 NQTKLPRVVGLTGVLIKGN-EITNVATKLKELEITYRGNIITVSDTKEMENVMLYATKPT

Tc_Dcr1 IQAPRPRILGLTAGLLGSEMQPDRLEAELQRLEKLLSSSVDTSS---EILTLIRLSCRPR

Dvv_Dcr1 PVFNRPRILGLTTGLLSSELQPDRLEGELRRLEKLLNSDVDTSS---EIVTLIRLCCRPH

Dm_Dcr1 PPADRPRILGLAGPLHSAGCELQQLSAMLATLEQSVLCQIETAS---DIVTVLRYCSRPH

Dm_Drosha PPAYGYEGYAYNE-------QAQKYGGQESHYQYQYPASGSSFL----------------

Tc_Drosha NFNVPPPPMG-------------HYNY-YPPYPAQ-------------------------

Dvv_Drosha PASYIPSSSGVT--------QQQHFDYRYSHLQSQYQTTGSVND----------------

. .

Tc_Dcr2 ELFKVCQPGALSLDAKQVLNNLRQLIN-----DLEHINIKDEQNSVNLLQSETLKPLEP-

Dvv_Dcr2 ERNVGFDRHSLDEDEVFVMTRLEKTIQ-----NLSSIKGNPMPITLSL---KGLVPIRA-

Dm_Dcr2 EVMVSFPH---QEQVLTVTRLISAEIEKFY-VSLDLMNIGVQPIR----RSKSLQCLRD-

Tc_Dcr1 ERIVECFKPIPSPLQDKIKATITSCQD-----FLKDHRYDPSEIY----DDDLLEEFKQV

Dvv_Dcr1 ETLIECPKNEPDSLYDEIKNIIKMAIE-----FLKDHRYDPSEIY----EDDLLEELKQV

Dm_Dcr1 EYIVQCAPFEMDELSLVLADVLNTHKS-----FLLDHRYDPYEIYG---TDQFMDELKDI

Dm_Drosha -----------------------------Y----ESYKYPD-------------------

Tc_Drosha ------------------------------------------------------------

Dvv_Drosha -----------------------------YTKDLENYRYIKSRVTET--ETEFIR-----

Tc_Dcr2 SDVLKSLRNLISDLMIHIEMLGAFGGHIACVAHMIQIERIKKHCQNHQLFIVLNYVMTIM

Dvv_Dcr2 EDGQKRLINLLKDLKYHVETMGIYGGSKACEVNIIKIERLKKHCEDMKLSMIFDSVQTTL

Dm_Dcr2 PSKKSFVKQLFNDFLYQMKEYGIYAASIAIISLIVEFDIKRRQAETLSVKLMHRTALTLC

Tc_Dcr1 PDPKEQPLSFFDDFLEILDDLGPWSADRAAYGMLIKIEKLKVKVPYERHYLLLCVASSVL

Dvv_Dcr1 PDPRTVPLELLEEFLEILKDLGPWGADKAALNILSKIEKLKVKIPYERHYLLLCMVSSAL

Dm_Dcr1 PDPKVDPLNVINSLLVVLHEMGPWCTQRAAHHFYQCNEKLKVKTPHERHYLLYCLVSTAL

Dm_Drosha ------------------------------------------------------------

Tc_Drosha ------------------------------------------------------------

Dvv_Drosha ------------------------------------------------------------

Tc_Dcr2 GTTKLLLEETMAG---------YEPLEKIRKFSSDKVLKV----FEILDEY-KTK-----

Dvv_Dcr2 SFITRKLNKRMED---------VSEKEKIYLYSSDQIKQL----IRIFNDYQQKK-----

Dm_Dcr2 EKIRHLLVQKLQDMTYDDDDDNVNTEEVIMNFSTPKVQRF----LMSLKVSFADK-----

Tc_Dcr1 VSIRALCELEFQD---------YTDKEKVFRFSTPKVLRF----LQVLKQFKPTGDKPET

Dvv_Dcr1 VTVRAVCDYVFES---------YDELERINKFSSPKVLKF----IDVLKQFKPPGEKPQP

Dm_Dcr1 IQLYSLCEHAFHRHL----GSGSDSRQTIERYSSPKVRRL----LQTLRCFKPEEVHTQA

Dm_Drosha -----------------------RYPAYSSNYRPPS-ERQRYTSNSSSQGYHHYPGYSS-

Tc_Drosha -----------------------DKPPKRYKDRPPPPSDY----SQELELYKQKKATNQ-

Dvv_Drosha ----------------------ISEKSRERKYRSPSPSRYRRSRSRDRDRYGRKRSVDK-

.

Tc_Dcr2 ------------------------------------------------------------

Dvv_Dcr2 ------------------------------------------------------------

Dm_Dcr2 ------------------------------------------------------------

Tc_Dcr1 CDKLPDLKD---------------------------------------------------

Dvv_Dcr1 SQLIKDKEETS-------------------------------------------------

Dm_Dcr1 DGLRRMRHQVDQADFNRLSHTLESKCRMVDQMDQPPTETRALVATLEQILHTTEDRQTNR

Dm_Drosha ------------------------------------------------------------

Tc_Drosha ------------------------------------------------------------

Dvv_Drosha ------------------------------------------------------------

Tc_Dcr2 --------------------------------------------SDEELCCLVFTKRRFT

Dvv_Dcr2 --------------------------------------------GTDEICCIVFTQRRST

Dm_Dcr2 --------------------------------------------DPKDICCLVFVERRYT

Tc_Dcr1 ---------------PKKGKGKNYKGP--RRPYISRAQ------SDEMLCALVFVKNRYK

Dvv_Dcr1 ---------------PVKIKCRG-KGP--RRPLFPRPQ------NEDNLCCLVFVHNRYK

Dm_Dcr1 SAARVTPTPTPAHAKPKPSSGANTAQPRTRRRVYTRRHHRDHNDGSDTLCALIYCNQNHT

Dm_Drosha -------------------------------------------------------GRRYE

Tc_Drosha -------------------------------------------------------RSRSP

Dvv_Drosha -------------------------------------------------------KRRYS

.

Tc_Dcr2 AKVLHHIIDKASQVDPKFY-HIKSNFVVGNKNNPYNDTRENLYITKKNREVLNSFVSKEI

Dvv_Dcr2 AQILFHILNALSQNYPQYS-YIKANFIVGHNSNPYNDTRELLYLHKKNKQILNDFTNKDI

Dm_Dcr2 CKCIYGLLLNYIQSTPELRNVLTPQFMVGR--NNISPDFESVLERKWQKSAIQQFRDGNA

Tc_Dcr1 AEALFALLCVMSKSDEEYW-WVSVSFSVNKIADPVREPREAESEHKRQEEVLRKYRSHEC

Dvv_Dcr1 AKSLFALLCSLSNVHDDLW-WISALFSVEKVADSRSEARQAENEHKTQEEVLRKFRCHEC

Dm_Dcr1 ARVLFELLAEISRRDPDLK-FLRCQYTTDRVADPTTEPKEAELEHRRQEEVLKRFRMHDC

Dm_Drosha QR------------------------------------------HDQEHRQIQDSRYAH-

Tc_Drosha R---------------------------------------------RRSRSPR-------

Dvv_Drosha R---------------------------------------------DRSRSRSSTRSRS-

.

Tc_Dcr2 NVLVSSNVLEEGVDIPKCTLVIKFDKSEDYRSYIQSKGR--------ARHIKSLYYTI--

Dvv_Dcr2 NVLVSSNVLEEGVDVPKCSLVIKYDCPMDYRSYVQSKGR--------ARHKESLYYVM--

Dm_Dcr2 NLMICSSVLEEGIDVQACNHVFILDPVKTFNMYVQSKGR--------ARTTEAKFVLF--

Tc_Dcr1 NIMIATSALEQGCDLPKCNLVIRFDLPQSFHSYIHSKAR--------ARANEAHFLLL--

Dvv_Dcr1 NILVSTSVLEQGCDLPKCNLVIRFDLPNTFYSYIQSKAR--------ARAPEAHYILF--

Dm_Dcr1 NVLIGTSVLEEGIDVPKCNLVVRWDPPTTYRSYVQCKGR--------ARAAPAYHVILVA

Dm_Drosha ------------------------EPRHGHYAHRQAKGSQHGYYGSAARNQVSDDYSPRG

Tc_Drosha --------------------------YSRYRKRSRSRSR---------------------

Dvv_Drosha ------------------------RGRSRYSQRRRSRSRSYEY----KRRRRSRTRTPSR

. :.:.

Tc_Dcr2 ---------------------------VETTDVA--------------------------

Dvv_Dcr2 ---------------------------VEVGTYR--------------------------

Dm_Dcr2 ---------------------------TADKERE--------------------------

Tc_Dcr1 ---------------------------ANENEVS--------------------------

Dvv_Dcr1 ---------------------------GNEQELD--------------------------

Dm_Dcr1 --PSYKSPTVGS-VQLTDRSHRYICATGDTTEADSDSDDSAMPNSSGSDPYTFGTARGTV

Dm_Drosha HHERERNETLEK-----TRAKP-----KVETERD--------------------------

Tc_Drosha -----------------PREKP------VK-ERD--------------------------

Dvv_Drosha GHSRYRSPSIKRSKKPTPSEIP------SESERD--------------------------

Tc_Dcr2 ------------------------------------------------------------

Dvv_Dcr2 ------------------------------------------------------------

Dm_Dcr2 ------------------------------------------------------------

Tc_Dcr1 ------------------------------------------------------------

Dvv_Dcr1 ------------------------------------------------------------

Dm_Dcr1 KILNPEVFSKQPPTACDIKLQEIQDELPAAAQLDTSNSSDEAVSMSNTSPSESSTEQKSR

Dm_Drosha -----RLLRQWCSNFC--------------------------------------------

Tc_Drosha -----LILSKWRKNYC--------------------------------------------

Dvv_Drosha -----QLLSKWRKNYC--------------------------------------------

Tc_Dcr2 ---------------------------------KYDKKYSAFKEIENLVNDLLIGKNSER

Dvv_Dcr2 ---------------------------------SFTQRVNGYKKIEDMLNQYLVGRNDER

Dm_Dcr2 ---------------------------------KTIQQIYQYRKAHNDIAEYLKDRVLEK

Tc_Dcr1 ---------------------------------DFVENLAEYNEVENTLLKRCYSLEPDK

Dvv_Dcr1 ---------------------------------KFVDNLAEYNEVENTLLRRCYSLEPNK

Dm_Dcr1 RFQCELSSLTEPEDTSDTTAEIDTAHSLASTTKDLVHQMAQYREIEQMLLSKCANTEPPE

Dm_Drosha -----------------------------EKPEDYVKKMNALSEAD--------------

Tc_Drosha -----------------------------STREEVSNKIHELAKVDH-------------

Dvv_Drosha -----------------------------STKQQLSDKLEELASMTV-------------

. .. .

Tc_Dcr2 DHPNLSEIRNMYNEDKLEPYYVNG--PNSAQVNMTSAVALLCRYCSNLASDKYTTYAPEW

Dvv_Dcr2 EEISEEDLQAMYNEDEIPPYFAK---PGGACVTMLSAISLLCQYCTSLSSDVYSDAAPEW

Dm_Dcr2 TEPELYEIKGHFQDD-IDPFTN----ENGAVLLPNNALAILHRYCQTIPTDAFGFVIPWF

Tc_Dcr1 NEELVADASSLQ----CRPYQPSAE-PGANSVSLSNAIALVNRYCAKLPSDTFTRLTPIW

Dvv_Dcr1 NEEMVADMYNNL----CKPYQPLSE-EGAPSITLINAISLLNKYCAKLPSDTFTRLTPIW

Dm_Dcr1 QEQSEAERFSAC----LAAYRPKPHLLTGASVDLGSAIALVNKYCARLPSDTFTKLTALW

Dm_Drosha -----APVESWVRSSPAELYYE----RTKSENEV-RGRARLQKLCTLF------------

Tc_Drosha EEVLEQEKNIWTRSTPSDLYYR----KDESNARVTRATKRLTQLCDKF------------

Dvv_Drosha EDILENEKNIWTRSTPAELYYQ----RDLEKPTVVNATQKLKQLCDEF------------

: . : : * :

Tc_Dcr2 YY--EED-------SSSAKLRVVIFLPVVCPLIDPIVGPYMHNKKDAKRAAALVACIKLH

Dvv_Dcr2 YK--KDS-------FEDGKVSVVILLPTGSHILDEIQGDYMGNIKLAKRAAAFRTCVLLH

Dm_Dcr2 HVLQEDERDRIFGVSAKGKHVISINMPVNCMLRDTIYSDPMDNVKTAKISAAFKACKVLY

Tc_Dcr1 H---EEK-------VENG-YICSIRLPINSPVKKTVTSPPMINTLLARRAAAFMICQLLH

Dvv_Dcr1 S---EDK-------TNDGKFICHLRLPINSPVKKTISSPPMTNGLLARRAAAFMICQLLH

Dm_Dcr1 RCTRNER-------AGVTLFQYTLRLPINSPLKHDIVGLPMPTQTLARRLAALQACVELH

Dm_Drosha ----DEE-------LLQRAKRVREKLPVYVP---------------PPRKARRRVCKHKH

Tc_Drosha ----NDC-------LVMRAAKVNKLKPKYEP---------------PPRKNRARLC--KH

Dvv_Drosha ----EEG-------LVTRARKVNAKKPKYVP---------------PPRKNRARLC--KH

.: * . * :

Tc_Dcr2 QC------------------------------------------------GELDNNLLPW

Dvv_Dcr2 QV------------------------------------------------GELDDHLLPV

Dm_Dcr2 SL------------------------------------------------GELNERFVPK

Tc_Dcr1 KA------------------------------------------------GELDDNLQPI

Dvv_Dcr1 MT------------------------------------------------KELDDYLQPI

Dm_Dcr1 RI------------------------------------------------GELDDQLQPI

Dm_Drosha KSEACSSSSSSDDDSDEDAFKIEQDCCMEELSRKVQHPQRVHADLWHNDAGEMNDG--PL

Tc_Drosha KSEESSSSGSSEEELTD-----EEDCTMEELQRKQQHPDRLHPEMWYNDPGEMNDG--PL

Dvv_Drosha KTEAQSSSDSSDADITD-----EEDCTMEELQRKQQHPDRLHPEMWFNEPGEMNDG--PL

*::: *

Tc_Dcr2 KKQ---LDEADVSYLFTHWPQEK--------ESDAGNKKKKRLHDKEIAPSVKSAI-QPD

Dvv_Dcr2 KKV---VKEEDVSFLFTHYPDKQ--------NKKNNSLKTRRLHKKMVPDALKEQI-RPS

Dm_Dcr2 T-----LKERVASIADVHFEHWNKYGDSVTATVNKADKSKDRTYKTECPLEFYDALPRVG

Tc_Dcr1 GKENFKVNEEDWNSSALEESDEE------NLDPRPGTTKRRQYYYKKVADALLDCHPIIG

Dvv_Dcr1 SKENFRATEDDWVNFALDESDDE------TSEVRPGTTKRRQYYYKRISEALLNCHPVAG

Dm_Dcr1 GKEGFRALEPDWECFELEPEDEQ--IVQLSDEPRPGTTKRRQYYYKRIASEFCDCRPVAG

Dm_Drosha CRCS--AKSRRIGIRHGIYPGETGYK---LCDPNSNNAGK--------------------

Tc_Drosha CRCS--IKSRKSGIRHGIYPGEKHLE---KCVPDSNNAER--------------------

Dvv_Drosha CRCS--LKSKKSGIRHGIYPGEQHLP---PCEMDSNNANR--------------------

.

Tc_Dcr2 RVLYLHTININPQYK-RSDDLKNAVTIYDLYKTP---LKFGLLSPKPLPDLCKFPLFDSN

Dvv_Dcr2 T-VYLHYLQLTPQFS-RHSLDINQSTLHDMYTSS---LSFGIITTSPLPTICDFPIYLTA

Dm_Dcr2 EICYAYEIFLEPQF----ESCEYTEHMYLNLQTP---RNYAILLRNKLPRLAEMPLFSNQ

Tc_Dcr1 QPTYFYKIVMKLTCPLPEEQNTRGRKIYPPEDSP---QGFGILTSKEIPKISAFPIFTRS

Dvv_Dcr1 QPTFFYKIMMTLTCPLPEEQNTRGRKIYPPEDSL---QGFGILTSKRIPKISAFPIFTRS

Dm_Dcr1 APCYLYFIQLTLQCPIPEEQNTRGRKIYPPEDAQ---QGFGILTTKRIPKLSAFSIFTRS

Dm_Drosha --LFHYRISISP----PTNFLTKTPTIIKHDEHEFLFEGFSLLSHVRLSDLPVCKVI---

Tc_Drosha --LYHYRITISP----PTNFLIKTPTIIHYDEHEFIFEGFSMFSHFPLEKLPTCKVI---

Dvv_Drosha --LFHYRITISP----PTNFLIKTPTIIHFDEHEFIFEGFSMFSHHPLEKLPNCKVI---

: : : : . : :.:: : : :

Tc_Dcr2 GTLEIEIRNNVREVEFAANE-MKEMREFHFLVFNDLLEILKEFLIFDNTGMNSEMLLVVP

Dvv_Dcr2 GTIQVSLKVNHGTLNITHAD-LEDIKTFHFTVFDSVLEILPRFLTLD-TSDDAEMMLLVP

Dm_Dcr2 GKLHVRVANAPLEVIIQNSEQLELLHQFHGMVFRDILKIWHPFFVLDRRSKENSYLVVPL

Tc_Dcr1 GEVSVDLQLCS-QLIVTENQ-ICKIREFLNYTFTSVLRLQKYLTLFNPDASANSYLIVPT

Dvv_Dcr1 GEVSVDLELISTDVVLSESQ-IEKTREFVNYTFTSVLRLQKYLMLFNPEASPNNYLIVPT

Dm_Dcr1 GEVKVSLELAKERVILTSEQ-IVCINGFLNYTFTNVLRLQKFLMLFDPDSTENCVFIVPT

Dm_Drosha -RFNIEYTIEYEEEKMPENFTIHELDIFFKYLFHELLELVDFNLMPN------------L

Tc_Drosha -RFNIEYTILYIEEKIPDNFTVRELDLFHDYLFREILELVDLDFK---------------

Dvv_Drosha -RFNIEYTILYIEERIPENFTVRELELFTEFLFHEILELVDLDFK---------------

. : . : * * .:*.:

Tc_Dcr2 VQDRCGDVCVDFRVIRDNKN--LKNKLEPAATERINLNVT-EETYLHKIVSPWYRSPPKM

Dvv_Dcr2 VDKERND--IDWGVLRSKVA--AQLVNELSEEQKINLEVT-QETHLNKIVNPWYRRDTAT

Dm_Dcr2 ILGAGEQKCFDWELMTNFRR--LPQSHGSNVQQREQQPAPRPEDFEGKIVTQWYANYDKP

Tc_Dcr1 I--DGATTTVDWDFIDLIYANLTVLPEIIPEEVRKSYEFD-PEKYRDAVVMPWYRNQDQP

Dvv_Dcr1 IKDDAQSVRVHWDFIDLIFNNLTTTPEFIVDEKRVDYEFK-AEAYSDAVVMPWYRNQDQP

Dm_Dcr1 VKAPAGGKHIDWQFLELIQANGNTMPRAVPDEERQAQPFD-PQRFQDAVVMPWYRNQDQP

Dm_Drosha PSGNVEESCPAFHFFPRFVR-------DLPDNGKEVLAMV--------EVLRYLLDNSAQ

Tc_Drosha AAGDV-DGCSQFHFMPRFVR-------ELPDNGKEILAMN--------EVLQYLLDSSVS

Dvv_Drosha AAGDK-EGCSQFHFLPRFVR-------ELPEKGKEILCMS--------QVLKYLLNSSVP

: .: : * :

Tc_Dcr2 --YVVTKVCPDKSALSRFP-NHEYPNFVSYYSEKHSLSI---LDPSQPLLLVKGLSERLN

Dvv_Dcr2 --YVVTEVCFNKSARSPFP-NESFSSFVEYFRSKHNITL---INPDLPLLRVKGLTKNRI

Dm_Dcr2 --MLVTKVHRELTPLSYMEKNQQDKTYYEFTMSKYGNRIGDVVHKDKFMIEVRDLTEQLT

Tc_Dcr1 QYFYVAEICSNLNPASDFP-GSDYATFEEYYLRKYSIQI---QNKSQHLLDVDHTSARLN

Dvv_Dcr1 QYFYVAEICSNLNPTSAFP-GSEYATFEEYYNRKYGIQI---QNLNQNLLDVDHTSARLN

Dm_Dcr1 QYFYVAEICPHLSPLSCFP-GDNYRTFKHYYLVKYGLTI---QNTSQPLLDVDHTSARLN

Dm_Drosha --LVERQQLLHLNQIS----QSEWQNYVDFIK---GMLV-TKPGYKPCSLRVDQLDRNNS

Tc_Drosha --LIEEKDLEDMIKMT----QYEWQSYADEIK---GMVV-TYPGKKPCSVRVDQLDRNID

Dvv_Drosha --LMKADDLGKMMEMT----QNEWQSYADEIK---GMVV-TYPGKKPCSVRVDQLDRNVD

. . : . .: . : . : * .

Tc_Dcr2 AFKPR-----------GAGGKRKKEKMYEELEEYLIPELVIKQEFPSCL-----------

Dvv_Dcr2 FIKPK-----------GASKGKRAYETNTELEEYLPPELVVKQEYPASL-----------

Dm_Dcr2 FY----VHNRG----------KFNAKSKAKMKVILIPELCFNFNFPGDL-----------

Tc_Dcr1 FLTPRYVNRKGVALPTSSEATKRAKREKLEQKQILVPELCAIHPFSASL-----------

Dvv_Dcr1 FLTPRYVNRKGVALPTSSEETKRAKRENLEQKQILVPELCAIHPFPGSL-----------

Dm_Dcr1 FLTPRYVNRKGVALPTSSEETKRAKRENLEQKQILVPELCTVHPFPASL-----------

Dm_Drosha DL-PECVDRET---------------------GISHPAIVHFGICHPQLSYAGNPEYQKA

Tc_Drosha ------LQKPG---------------------DYKFPEIVHFGIRPPQLSYAGNPDYQKA

Dvv_Drosha ------EQIEG---------------------NYTFPEIVHFGIRPPQLSYAGNPDYQKA

* : *

Tc_Dcr2 WIQARFLPSILSRLAYLLKLQQLQVDIARGIGAKAEYLK----DCPPLELNLHLLHY---

Dvv_Dcr2 WIQSSFMPTILSRLSFMLQLEQLRCTIVQETGMGSVIHN----TRKPLELDEYLLDY---

Dm_Dcr2 WLKLIFLPSILNRMYFLLHAEALRKRFNTYLNLHLLPFNGTDYMPRPLEIDYSLKRNV--

Tc_Dcr1 WRKAVCLPCILYRINALLLADQIRRTVALELNLGKIELD-SEFKWPPLNFGWSLADVLKK

Dvv_Dcr1 WRKAVALPCILYRINALLLADQIRRTVAEALRLGKSNLS-EDFKWPSLNFGWSLSDVLKK

Dm_Dcr1 WRTAVCLPCILYRINGLLLADDIRKQVSADLGLGRQQIEDEDFEWPMLDFGWSLSEVLKK

Dm_Drosha WREYVKYRHLMANMS---------------------------------------------

Tc_Drosha WRDYVKFRHLLANMS---------------------------------------------

Dvv_Drosha WRDYVKFRHLLANMS---------------------------------------------

* :: .:

Tc_Dcr2 -------------------------EPNDPQLTQESDKSTPL-IDNCLA-LECPKNLRTI

Dvv_Dcr2 -------------------------KPYVEEIPHDED--TPMDIDQVDA-VQALPLNAVS

Dm_Dcr2 -------------------------DPLGNVIPT-ED------IEEPKSLLEPMPTKSIE

Tc_Dcr1 SKDEE-KKKQE-------KIEPVIEEIPCTEIAKIEDFDQ---DDD--------------

Dvv_Dcr1 SREEELRKKQENLAALETKVTEKLQTLTIREIKSDEDPES---IDDCSN-DDTKLETYIS

Dm_Dcr1 SRESKQKESLKDDTINGKDLADVEKKPTSEETQLDKD-----------S-KDDKVEKSAI

Dm_Drosha -------------------------KPSF------KDKRK---LEEKEQRLQEMRTQGRM

Tc_Drosha -------------------------KPTF------EDKRK---LESKENKLQEMRTQGKM

Dvv_Drosha -------------------------KPTY------EDKRK---LEAKENKLQEMRTQGKM

.*

Tc_Dcr2 QYNKDFAAK------MLEAEYYWK------------------------------------

Dvv_Dcr2 RINKQFTNK------IIESQYPWK------------------------------------

Dm_Dcr2 ASVANLE--------ITEFENPWQ------------------------------------

Tc_Dcr1 ------EEE------MIEI-GTWSNDMA-------QLNSDQE----FPVV----------

Dvv_Dcr1 DSNNEGESK------WIEI-GTWCNEMA-------GSTDPEN----SAVV----------

Dm_Dcr1 ELIIEGEEKLQEADDFIEI-GTWSNDMADDIASFNQEDDDEDDAFHLPVLPANVKFCDQQ

Dm_Drosha KRNITV---------AISSEGFYRTGIM------------------CDVV---------Q

Tc_Drosha KRDITV---------AVSAEGFYRTGIM------------------CDII---------Q

Dvv_Drosha KRDVTV---------AVSAEGFYRTGIM------------------CDII---------Q

. :

Tc_Dcr2 -TIEEPK--------------------------------------DIERNINVTV-MDIE

Dvv_Dcr2 -DIEEPL--------------------------------------DVERDLNATV-LDVE

Dm_Dcr2 -KYMEPV--------------------------------------DLSRNLLSTYPVELD

Tc_Dcr1 -RYASPTSWMDLQN----------------------------TYDDSSFSDSDYSGDESE

Dvv_Dcr1 -RYASPTSWMAVDT----------------------------NYEDFSDSESDMYDEDSD

Dm_Dcr1 TRYGSPTFWDVSNGESGFKGPKSSQNKQGGKGKAKGPAKPTFNYYDSDNSLGSSYDDDDN

Dm_Drosha HAMLIPV---------------------------------LTGHLRFHKSLDLLE-ESIG

Tc_Drosha HAMLIPV---------------------------------LVCHLRFHHSLNILE-ESVN

Dvv_Drosha HAMLIPV---------------------------------LVCHLRFHHSLNVLE-EKVK

* . .

Tc_Dcr2 Y-----------------------------------------------------------

Dvv_Dcr2 H-----------------------------------------------------------

Dm_Dcr2 Y------------------------YYHFSVGNVCEMNE---------MDFE--------

Tc_Dcr1 S-------------------EWGGLRIEFTGDNVAEAVDD---ENKKDDDFELVDYSNVW

Dvv_Dcr1 T-------------------ELGPFRIEFTGDHQAEAVDDE--EDEEDSSFKLYNDDSVW

Dm_Dcr1 AGPLNYMHHNYSSDDDDVADDIDAGRIAFTSKNEAETIETAQEVEKRQKQLSIIQATNA-

Dm_Drosha Y--------------------------RFKNRYLLQLALTHP------------------

Tc_Drosha Y--------------------------KFKNRALLQLALTHP------------------

Dvv_Drosha Y--------------------------KFKNRALLQLALTHP------------------

Tc_Dcr2 ------------------------------YETFISHQPSK--TGRLLKNDS--------

Dvv_Dcr2 ------------------------------YEQFLGFKLTP--STRELKNTS--------

Dm_Dcr2 ------------------------------DKEYWAKNQFHMPTGNIYGNRT--------

Tc_Dcr1 KVEDESEITQTL----------RKQ-----FHDACARNKDHILSSGILVSKSEQFQKCSD

Dvv_Dcr1 QIDEENELTENL----------KKE-----FHAACDKNKKHILSSGILIKRDSTFSKHSS

Dm_Dcr1 -NERQYQQTKNLLIGFNFKHEDQKEPATIRYEESIAKLKTEIESGGMLVPHDQQLVLKRS

Dm_Drosha -----------------------------SYKENYGTNPDH--ARNSLTNCG--------

Tc_Drosha -----------------------------SYRENFGTNPDH--ARNSLTNCG--------

Dvv_Drosha -----------------------------SYKENFGTNPDH--ARNSLTNCG--------

. :

Tc_Dcr2 ------------------------PVK-QQNVPAITYDC---------------------

Dvv_Dcr2 ------------------------PIK-RKELLALTYDK---------------------

Dm_Dcr2 ------------------------PAKTNANVPALMPSK---------------------

Tc_Dcr1 CDNTTTKDSQVANSYDFDFGKLFIELD-QHKALQIDLAPQDARNEYDISETMTFKFD---

Dvv_Dcr1 RDK---------------------------EGVTVFLNP--IVNNIDLSGLYMTPIE---

Dm_Dcr1 DAA----EAQVAKVSMMELLKQLLPYV-NEDVLAKKLGD---RRELLLSDLVELNADWVA

Dm_Drosha -------IRQ--------------PEYGDRKIHYMNTRK---------------------

Tc_Drosha -------IRQ--------------PEYGDRRIHYMNTRK---------------------

Dvv_Drosha -------IRQ--------------PEYGDRRIHYMNTRK---------------------

Tc_Dcr2 -------------------------QFEAKQLQIL----------------------DVQ

Dvv_Dcr2 -------------------------SFIPTKINLL----------------------EVQ

Dm_Dcr2 -------------------------PTVRGKVKPL-------------------LILQKT

Tc_Dcr1 ----EQPNLVEHPGPSPNLHQHKALQI---DLAPQDARNEYDIS----------ETMTFK

Dvv_Dcr1 ----QQMD----------------------DILELEYFNEYDKS----------EPLSFS

Dm_Dcr1 RHEQETYNVMGCGDSFDNYNDHHRLNLDEKQLKLQYERIEIEPPTSTKAITSAILPAGFS

Dm_Drosha -----------------------------RGINTL-------------------VSIMSR

Tc_Drosha -----------------------------RGINTL-------------------INIMSR

Dvv_Drosha -----------------------------RGINTL-------------------INIMSR

:

Tc_Dcr2 FDNQ-------SP-NLCQIYQALTAAEANDIVNLERLETLGDSFLKFVASLYIIFKFPTY

Dvv_Dcr2 PDEK-------GP-ELCEFYRAFTCAKANDIVNLERLETLGDSFLKLISSVYIYLKFPKY

Dm_Dcr2 VSKE-----HITPAEQGEFLAAITASSAADVFDMERLEILGDSFLKLSATLYLASKYSDW

Tc_Dcr1 FDEQPNLVEHPGP-SPNVLLQALTMSNANDGINLERLETIGDSFLKYAITNYLYSKYENV

Dvv_Dcr1 FDKQPNLEEHPGP-SPNVLLQALTMSNANDGINLERLETIGDSFLKYAITNYLYSKYENV

Dm_Dcr1 FDRQPDLVGHPGP-SPSIILQALTMSNANDGINLERLETIGDSFLKYAITTYLYITYENV

Dm_Drosha FGKE--------------------HETVSNITHNERLEFLGDAVVEFLSSIHLFFMFPEL

Tc_Drosha FGKQ--------------------QETESNITHNERLEFLGDAVVEFLSSIHLFYTFPDL

Dvv_Drosha FGKQ--------------------QETESNITHNERLEFLGDAVVEFLSSIHLFYSFPDL

..: : . **** :**:.:: : :: :

Tc_Dcr2 NEGKSTTLKGKLVSNKNLYYLGVRKNLGGILKNSDLSPS-DWVPPCFCIPQTISKAIGNK

Dvv_Dcr2 NEGLATALKGRLVSNKNLLYLAMRKNLQGIIKYHELSPQKEWLPPAFTIPSELSQRIEDK

Dm_Dcr2 NEGTLTEVKSKLVSNRNLLFCLIDADIPKTLNTIQFTPRYTWLPPGISLPHNVLALWREN

Tc_Dcr1 HEGKLSHLRSKQVSNLNLYRLGRRKGLGEYMIATKFDPHDNWLPPCFYVPKELEEALIDA

Dvv_Dcr1 HEGKLSHLRSKQVSNLNLYRLGRRKYLGEFMIATKFDPHDNWLPPCFYVPKQLEEALIDA

Dm_Dcr1 HEGKLSHLRSKQVANLNLYRLGRRKRLGEYMIATKFEPHDNWLPPCYYVPKELEKALIEA

Dm_Drosha EEGGLATYRAAIVQNQHLALLAKKLQLEEFMLYAHGS-----------------------

Tc_Drosha EEGGLATYRAAIVQNQHLAVLAKTLKLDQFMLYAHGS-----------------------

Dvv_Drosha EEGGLATYRAAIVQNQHLSVLAKILNLDQYMLYAHGS-----------------------

.** : :. * * :* : : .

Tc_Dcr2 EY-----SVVSLFNCCISPEEQV----SGNLNRKTLSDMTTEEIA-------PD--EENS

Dvv_Dcr2 EL-----SVSAVFGFTIPMEEQI----SGVLSEETKQEIAEENYP-------PSNTEESA

Dm_Dcr2 PEFAKIIGPHNLRDLALGDEESLVKGNCSDINYNRFVEGCRANG--------QSFYAGAD

Tc_Dcr1 QFPANCWTVADMAATRDMTLDDI----CSMVRQRGESLSLSN------------------

Dvv_Dcr1 QYPANCWSVADMAATRNMSLDDI----CLMVRERAEGYTLTSN-------------LLSD

Dm_Dcr1 KIPTHHWKLADLLDIKNLSSVQI----CEMVREKADALGLEQNGGAQNGQLDDSNDSCND

Dm_Drosha ---------------------DL----CHEL-----------------------------

Tc_Drosha ---------------------DL----CHDL-----------------------------

Dvv_Drosha ---------------------DL----CHDL-----------------------------

.: . :

Tc_Dcr2 YGNMC--NF-LNKQYVGDKSIADSVEALLGAYFLSGGIQGGIKFMEWIGI--LPLSEQI-

Dvv_Dcr2 YQTMA--NF-IKCQYIGDKFIADVVESLIGTYLRWGGFKGGIRIMEWIGI--IPKSERL-

Dm_Dcr2 FSSEV--NFCVGLVTIPNKVIADTLEALLGVIVKNYGLQHAFKMLEYFKICRADIDKPL-

Tc_Dcr1 ---IIPYNL-VTQHSIPDKSIADCVEALIGAYLIECGPRGALLFMAWLGIRVLP------

Dvv_Dcr1 GPMAIPYNL-VTQHSIPDKSIADCVEAVIGAYLIECGPRGALLFMAWLGIRVLP------

Dm_Dcr1 FSCFIPYNL-VSQHSIPDKSIADCVEALIGAYLIECGPRGALLFMAWLGVRVLPITRQLD

Dm_Drosha -------EL--------RHAMANCFEALMGALLLDGGIKVADEVFTDALFRQDE---KL-

Tc_Drosha -------EL--------RHAMANCFEALMGALFLDGGINVVDRVFSETLFKVNP---DL-

Dvv_Drosha -------EL--------RHAMANCFEALMGALFLDGKIEIADMVFSATYFHDKP---EL-

:: : :*: .*:::*. . . .: .

Tc_Dcr2 -----QRLIE----------------TTQVDPV-----LNKKSTKTDVDFHMPQWREIEQ

Dvv_Dcr2 -----EYMWQ----------------QPSPDPLL--LPLSQHELNEKIRHHLPLCSKIEQ

Dm_Dcr2 -----TQLLNLELGGKK---------------------MRANVNTTEIDGFLINHYYLEK

Tc_Dcr1 ------QLED----------GTYGEIELPKSPLS----NHLTYPREELDMLLDGYDQFER

Dvv_Dcr1 ------RLPD----------DGYGEIKPPRSPLL----RHVHDPEGELTRLLDGYDKFER

Dm_Dcr1 GGNQEQRIPGSTKPNAENVVTVYGAWPTPRSPLL----HFAPNATEELDQLLSGFEEFEE

Dm_Drosha -----LSIWK----------------NLPEHPLQEQEPLGDRSCIDSYR-VLKELTKFED

Tc_Drosha -----LEVWM----------------NLPPHPLQEQEPTGDREWIPKFE-LLQNLTKFEE

Dvv_Drosha -----FEVWA----------------NLPPHPLQEQEPLGDRKWIEKFD-ILQNLVKFEE

: . : :*

Tc_Dcr2 RLGYTFTNRAFLLQALTHSSYSPNRIT-LSYERLEFLGDAVLDFLITCYIFEHCGHLEPG

Dvv_Dcr2 ILGYRFNNKAYLLQALTHNSYTPNRTT-LSYQKLEFLGDAVLDFLITCHIYETCENLDPG

Dm_Dcr2 NLGYTFKDRRYLLQALTHPSYPTNRIT-GSYQELEFIGDAILDFLISAYIFENNTKMNPG

Tc_Dcr1 HIGYKFRDRSYLLQALTHASFSPNTLT-DCYQRLEFLGDAVLDYLITRHLYEDTRMHSPG

Dvv_Dcr1 HIGYKFADRSYLLQAMTHASYFPNHLT-DCYQRLEFLGDAVLDYLITRHLYEDPRMHSPG

Dm_Dcr1 SLGYKFRDRSYLLQAMTHASYTPNRLT-DCYQRLEFLGDAVLDYLITRHLYEDPRQHSPG

Dm_Drosha SIGIKFKHIRLLARAFTDRSIGFTHLTLGSNQRLEFLGDTVLQLICSEYLYRHFPEHHEG

Tc_Drosha SVGLQFNHIRLLARAFTDRSVGYTNLTLGSNQRLEFLGDTVLQLIASEYLYKYFPEHHEG

Dvv_Drosha SINVEFKHIRLLARAFTDRSVGYTNLTMGSNQRLEFLGDTVLQLIASDYLYKYFPEHHEG

:. * . * :*:*. * . * . :.***:**::*: : : :::. *

Tc_Dcr2 QVTDLRSSLVNNNTFASLVVRCGFHKFLLMMNSNLQGHIDKFADYLASKNYVIDDEVLIL

Dvv_Dcr2 QLTDLRSALVNNNTFASLCVRLRLHTYLLMMNMQLQKLIDRFDNFMAQKNYIIDDEVLIL

Dm_Dcr2 ALTDLRSALVNNTTLACICVRHRLHFFILAENAKLSEIISKFVNFQESQGHRVTNYVRIL

Tc_Dcr1 ALTDLRSALVNNTIFASLAVRNGFHRYFRNLSPSLNEVVEKFVRLQEDSGHTLVDELYLV

Dvv_Dcr1 ALTDLRSALVNNTIFASLAVRNNFHKYFRHLSPGLNEVVERFIRLQEESGHSLVEELYLV

Dm_Dcr1 ALTDLRSALVNNTIFASLAVRHGFHKFFRHLSPGLNDVIDRFVRIQQENGHCISEEYYLL

Dm_Drosha HLSLLRSSLVNNRTQAVVCDDLGMPKYAVYANPKAD------------------------

Tc_Drosha HLSLLRSSLVNNRTQAVVCDDLGMSNYAVYNNPKAE------------------------

Dvv_Drosha HLSLLRSSLVNNRTQAVVCDDLDMAKYAIYNNPKAE------------------------

:: ***:**** * : : : . .

Tc_Dcr2 LEE-------------------------------------DEMNIAEYVDVPKVLGDIFE

Dvv_Dcr2 LQE-------------------------------------DEYQLAESVDVPKVLGDIFE

Dm_Dcr2 LEEADVQPTPLDLDDELDMTELPHANKCISQEAEKGVPPKGEFNMSTNVDVPKALGDVLE

Tc_Dcr1 VET----------------------------------------EEVEDVEVPKALGDVFE

Dvv_Dcr1 VET--------------------------------------ECEEIEDVEVPKALGDVFE

Dm_Dcr1 SEE--------------------------------------ECDDAEDVEVPKALGDVFE

Dm_Drosha LKT-------------------------------------------------KDRADLLE

Tc_Drosha LKT-------------------------------------------------KDRADLLE

Dvv_Drosha LKT-------------------------------------------------KDRADLLE

: * .*::*

Tc_Dcr2 ALAGAIYLDSNKDLKTVWRVFYKIIWREIDLFSKNV----PKNVI-------RRLYECHT

Dvv_Dcr2 AVAGAIYLDSKKCLKTVWEVFYKIMWKEISLFSSNI----PKNAI-------RRLFEWTS

Dm_Dcr2 ALIAAVYLDC-RDLQRTWEVIFNLFEPELQEFTRKV----PINHI-------RQLVEHKH

Tc_Dcr1 SVAGAIFLDSGMSLDAVWKVYYNMMKSEIEQFSNKV----PKSPI-------RELLELEP

Dvv_Dcr1 SVAGAIFLDSGMSLDAVWKVYYKMMKPEIEQFSNKV----PKSPI-------RELLELEP

Dm_Dcr1 SIAGAIFLDSNMSLDVVWHVYSNMMSPEIEQFSNSV----PKSPI-------RELLELEP

Dm_Drosha AFLGALYVD--KGLLYCEQFCHVCLFPRLQLFIMNQDWNDPKSKLQQCCLTLRTMDGGEP

Tc_Drosha AFIGALYVD--QGLEFCEVFCQVTLFPRLQDFIMNQDWNDPKSKLQQCCLTLRTMDGGEP

Dvv_Drosha AFIGALYVD--QGMEFCEVFCQVTLFPRLQDFIMNQDWNDPKSKLQQCCLTLRTMDGGEP

:. .*:::* : . : .:. * . * . : * :

Tc_Dcr2 VYPPQFSKAL---EVGNQKTMVSLDFMCEGRKKRVHGFGTNKILAKRAAAKIALR-----

Dvv_Dcr2 AH-PKFGKTV---PTENKKTMIPLRFQLNGIPTVVHGFGTNTAMAKKAAAKLALQ-----

Dm_Dcr2 AK-PVFSSP----IVEGETVMVSCQFTCMEKTIKVYGFGSNKDQAKLSAAKHALQ-----

Tc_Dcr1 ET-AKFGKPEK--LADGRRVRVTVEVFGKG---VFKGIGRNYRIAKCTAAKCALK-----

Dvv_Dcr1 ET-AKFGKPEK--LADGRRVRVTVEVFGKG---LFKGIGRNYRIAKCTAAKCALK-----

Dm_Dcr1 ET-AKFGKPEK--LADGRRVRVTVDVFCKG---TFRGIGRNYRIAKCTAAKCALR-----

Dm_Drosha DI-PYYKVVEASGPTNTRVYKVAVYFRSKR---LATSSGSSIQQAEMNAAKQALENSRDL

Tc_Drosha DI-PVYKSVVC--FTNTRVYTVAVYFRGRR---LASAMGHSIQQAEMNAAKKALEISQDL

Dvv_Drosha DI-PVYKVIECKGPTNTRVYTVAVYFRGRR---LASAMGHSIQQAEMNAAKNALEISHDL

. : . . :. . . * . *: *** **.

Tc_Dcr2 --ALKL------------------------------------------------------

Dvv_Dcr2 --LLLER-----KDKK--------------------------------------------

Dm_Dcr2 --QLSK----------CDA-----------------------------------------

Tc_Dcr1 --NLKKRGLIKKIDES--------------------------------------------

Dvv_Dcr1 --HLKRRGLLRKVEN---------------------------------------------

Dm_Dcr1 --QLKKQGLIAKKD----------------------------------------------

Dm_Drosha FPQLDHQKRVIAKSIKKQT-GNELDNDSDRQHQEEKIKRPKYATPLQDESHLPKQYRMHE

Tc_Drosha FPQLDHQKRVIAKSMKKQKNKKRRRSRSRSGLKF--------------------------

Dvv_Drosha FPQLDHQKRVIAKSMKKQK-KGRSVSKSRSEQSA--------------------------

*

Tc_Dcr2 ------------------------------------------------------------

Dvv_Dcr2 ------------------------------------------------------------

Dm_Dcr2 ------------------------------------------------------------

Tc_Dcr1 ------------------------------------------------------------

Dvv_Dcr1 ------------------------------------------------------------

Dm_Dcr1 ------------------------------------------------------------

Dm_Drosha NISSDELPEDEDFESTAPKSPTMPLRSNRSGSSSSSSSSDSDGSTSSPKCKRRLKKCSSV

Tc_Drosha -----------GFRTRSPESYSRRRFQRSRSRSKVTSESDSSPRCSTSKAKAYL------

Dvv_Drosha --------------PKSPKRYSRSRFKRSSSMSSSSE-----------------------

Tc_Dcr2 --------

Dvv_Dcr2 --------

Dm_Dcr2 --------

Tc_Dcr1 --------

Dvv_Dcr1 --------

Dm_Dcr1 --------

Dm_Drosha SSKSSLG-

Tc_Drosha -STEDLSD

Dvv_Drosha --------
